# The Genomic Equity Accessibility Framework (GEAF): A dimensionally consistent framework for modeling affordability and equity in genomic and regenerative therapies

**DOI:** 10.1371/journal.pone.0359782

**Published:** 2026-10-06

**Authors:** Peter Oloche David

**Affiliations:** Strayer University, Washington, District of Columbia, United States of America; Huazhong Agriculture University, CHINA

## Abstract

**Background:**

Advances in CRISPR-based gene editing and stem cell-derived embryo models have created substantial biomedical opportunity alongside significant ethical and distributive concerns. Personalized genomic and regenerative therapies approved in the United States as of this writing carry list prices spanning approximately $2.2 million (Casgevy) to $4.25 million (Lenmeldy), with several other approved products priced between these figures (e.g., Lyfgenia, $3.1 million; Hemgenix, $3.5 million). This raises the question of how affordability, accessibility, ethical compliance, and distributional inequality can be jointly represented in a single, mathematically coherent index to support policy analysis.

**Methods:**

We audited a previously proposed composite index, the Genomic Equity Accessibility Framework (GEAF = ((A x E) – (C x I))/(P + R) x 100), and identified dimensional incompatibility between its terms, an unbounded and not-independently-interpretable output range, and scenario inputs inconsistent with the cost estimates cited elsewhere in the same manuscript. We reconstructed GEAF as a normalized, bounded (0–100) weighted composite of six dimensionless components – accessibility, ethical compliance, affordability, equity (inverse inequality), policy strength, and research efficiency – derived using multi-attribute utility theory. We tested the reconstructed formula against algebraic edge cases, applied it to seven re-specified illustrative scenarios whose cost inputs are anchored to cited list-price literature, and conducted one-way, two-way, and Monte Carlo (10,000-iteration) probabilistic sensitivity analyses.

**Results:**

The reconstructed index (GEAF’) is bounded on [0,100], monotonic in every component, and behaves consistently at algebraic extremes. Across the seven illustrative scenarios, GEAF’ ranged from 27.9 (no equity intervention, list-price cost) to 86.5 (fully optimized tiered-pricing and policy scenario). One-way sensitivity analysis identified cost (C) and the inequality factor (I) as the two largest single-parameter drivers of the index under baseline conditions, followed by ethical compliance (E). Monte Carlo simulation (10,000 iterations per regime) propagating stated, non-empirical parameter-uncertainty assumptions produced a baseline-scenario mean of 32.6 (90% interval, 5th-95th percentile, approximately 22.9–42.9) and an equity-intervention-scenario mean of 72.4 (90% interval approximately 62.9–81.1), with no overlap between the two simulated distributions.

**Conclusions:**

GEAF’ provides a transparent, reproducible, and dimensionally coherent way to combine affordability, accessibility, ethical compliance, and distributional equity into a single comparative index for genomic and regenerative therapy policy scenarios. It is presented as a candidate conceptual and methodological framework, not as an empirically validated predictive or clinical tool; quantitative claims about real-world cost reductions have been removed unless directly supported by cited evidence. Prospective empirical calibration against real accessibility, pricing, and policy-outcome data is identified as a necessary next step before GEAF’ is used in health technology assessment or resource-allocation decisions.

## 1. Introduction

The maturation of CRISPR-Cas9 gene editing and of stem cell-derived synthetic embryo models (e.g., blastoids and gastruloids) has expanded what is technically possible in human genomics and regenerative medicine. Approved gene therapies now exist for conditions such as sickle cell disease, transthyretin amyloidosis, and spinal muscular atrophy [1], and dozens more are in late-stage development. These advances raise two distinct but related problems that this paper addresses in different ways. The first is ethical: non-therapeutic genetic enhancement, the moral status of increasingly sophisticated embryo models, and the governance of stem cell research raise questions that are the subject of an active bioethics and policy literature, which this manuscript summarizes rather than attempts to resolve. The second is distributive: even where a therapy is technically approved and effective, list prices ranging from approximately $2.2 million (Casgevy, Vertex [2] to $4.25 million (Lenmeldy, Orchard [3] with Lyfgenia priced at $3.1 million [4] and Hemgenix at $3.5 million [5] place it outside the reach of most of the world’s population, and outside many health systems entirely. The projected growth in gene-therapy spending also highlights the potential budgetary implications of these treatments [6]. Real-world evidence from Europe and the United States further highlights the importance of examining gene therapy access and outcomes beyond regulatory approval [7].


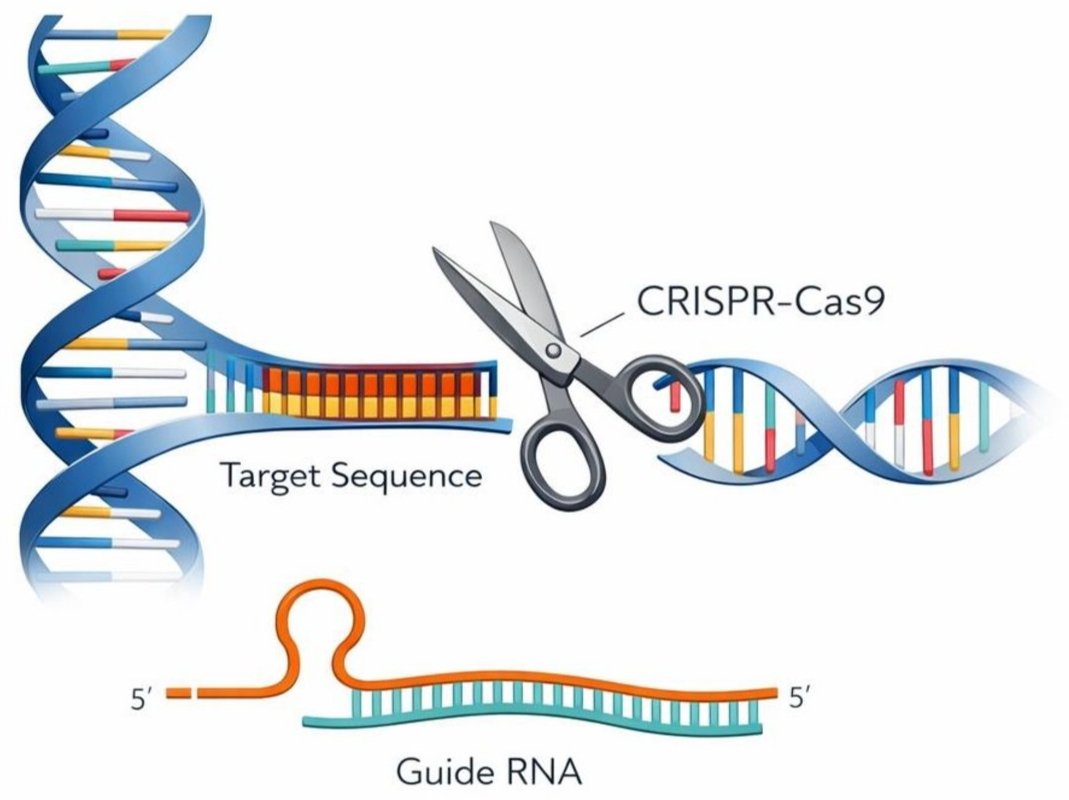


A single mathematical index, the Genomic Equity Accessibility Framework (GEAF), was previously proposed to combine affordability, accessibility, ethical compliance, and inequality into one number that could support policy comparison. That original formulation had several well-founded problems: the terms being combined were not on comparable scales (a percentage multiplied against a probability, subtracted from a cost in hundreds of thousands multiplied by a coefficient), the resulting output had no defined or defensible range, and the illustrative scenario inputs were not traceable to any stated empirical source. These defects could not be resolved by re-deriving weights or re-normalizing the existing expression in place; the index was instead reconstructed from first principles, and this manuscript reports the properties of the reconstructed version under systematic testing.

### 1.1. Research questions and objectives

This paper is framed as a methodological/framework contribution rather than an empirical clinical study. It addresses four research questions:

RQ1. Can affordability, accessibility, ethical compliance, and distributional inequality be combined into a single composite index that is dimensionally consistent, bounded, and monotonic in each component?

RQ2. Which parameters of such an index have the greatest influence on its output, and how does the index behave under one-way, two-way, and probabilistic (Monte Carlo) sensitivity analysis?

RQ3. How does a reconstructed composite index of this kind relate conceptually to established health-economic and equity-weighting frameworks (e.g., incremental cost-effectiveness ratios, distributional cost-effectiveness analysis, extended cost-effectiveness analysis)?

RQ4. What empirical data would be required to move such an index from an illustrative, assumption-based tool to one that is empirically calibrated and prospectively validated?

The primary objective is to reconstruct GEAF into a mathematically defensible form (RQ1-RQ2) and to characterize its behavior transparently (RQ2). A secondary objective is to position the reconstructed index relative to existing frameworks (RQ3) and to specify, rather than claim to have completed, the empirical validation agenda implied by RQ4. Because no primary data were collected, no formal statistical hypotheses are tested; the study is exploratory and conceptual, consistent with a modeling/methods paper.

### 1.2. Scope and unit of analysis

The unit of analysis for GEAF’ is a single named therapy-jurisdiction-policy scenario (e.g., “gene therapy X, under policy configuration Y, in setting Z”) evaluated at one point in time. This is distinct from a patient-level outcome measure (such as a QALY) and from a purely economic measure (such as an ICER); GEAF’ is a comparative policy-scenario index, not an individual-level clinical or actuarial metric. This distinction is maintained throughout the manuscript and is restated in the Discussion and Limitations.

## 2. Conceptual background: Ethical and governance context

This section summarizes, without attempting to resolve, the ethical and governance debates that motivate the equity problem GEAF’ is intended to help quantify. It is intentionally concise, focusing only on material that connects directly to the quantitative model and to the governance concepts introduced in Table 1. Readers seeking a full treatment of the underlying bioethics literature are directed to the cited primary sources.

**Table 1:** Proposed conceptual governance mechanisms (explicitly non-binding, non-existing institutions).

Mechanism	Purpose	Membership/Scope	Decision process	Institutional status	Limitations/ open questions
Ethical Enhancement Councils (EECs)	Case-by-case ethical review of proposed non-therapeutic gene-editing applications	National/regional deliberative bodies with scientist, ethicist, patient-advocate, and community representation	Structured multi-criteria deliberation (harm, consent, reversibility, social impact); decisions and dissents published	Advisory to national regulators; no binding international authority proposed	Open question flagged for future work: composition weighting and conflict-of-interest rules are not yet specified
Tiered Moral Framework (TMF)	Graduated ethical review scaled to developmental stage of synthetic embryo models	Applies to researchers and institutional review boards working with SCBEMs	Pre-implantation-equivalent models: minimal review; post-gastrulation-equivalent models: mandatory independent ethics review; models approaching neurulation-equivalent organization: prohibited pending further international guidance	Consistent with, but more granular than, ISSCR [8] SCBEM review pathway	Open question flagged: operational criteria for “post-gastrulation-equivalent” staging in vitro are not yet standardized across labs
International Stem Cell Accords (ISCAs)	Non-binding international commitment to shared ethical standards, data transparency, and benefit-sharing	Modeled conceptually on environmental treaty structures (e.g., Cartagena Protocol [9]); no existing treaty text	Voluntary reporting plus peer review among signatory states/institutions	Aspirational; would require formal diplomatic negotiation not undertaken in this paper	Explicitly not an existing agreement; presented as a policy proposal only

### 2.1. Non-therapeutic genetic enhancement

CRISPR-based editing capable of correcting single-gene disease can, in principle, also be directed at non-therapeutic trait modification (“designer babies”) [10]. Proponents invoke procreative beneficence [11]: a consequentialist position holding that parents have a defeasible reason to select for a child’s expected wellbeing where feasible. Critics invoke deontological concerns about instrumentalizing future persons and about the historical association between trait-selection ideology and eugenics [12], and note the discriminatory potential of widening access gaps for enhancement rather than treatment. The 2018 He Jiankui case, in which embryos were edited for HIV resistance outside ethical review and international norms, remains the primary real-world illustration of the treatment/enhancement boundary being crossed without adequate oversight [13,14].

### 2.2. Moral status of synthetic embryo models

Stem cell-derived embryo models (blastoids, gastruloids) recreate aspects of early human development without fertilization, providing an alternative to donated embryos for developmental and infertility research [15]. Their increasing structural fidelity to natural embryos raises the classical question of when, if at all, protections tied to potentiality or developmental stage should apply. The [8] guideline update retained the 14-day limit on integrated culture for natural embryos while introducing a specific, more flexible review pathway for stem cell-based embryo models (SCBEMs), rather than either abolishing or mechanically extending the 14-day rule [8]. This is a narrower and more specific claim than the earlier framing of this guideline, and is described accordingly here.

### 2.3. Regulation of stem cell research

Regulatory stringency for embryonic and induced pluripotent stem cell (iPSC) research varies substantially across jurisdictions, and the ISSCR guidelines function as an influential but non-binding international reference point rather than enforceable law [8,16]. Persistent governance gaps include inconsistent oversight of “stem cell tourism,” unclear consent standards for donated biological material, and the absence of a binding international mechanism comparable to environmental treaties. Australia’s National Health Genomics Policy Framework provides an example of a national policy approach to genomics governance [17].

### 2.4. Proposed governance concepts (explicitly conceptual, not existing institutions)

Three governance mechanisms are proposed here - Ethical Enhancement Councils (EECs), a Tiered Moral Framework (TMF), and International Stem Cell Accords (ISCAs) – and are operationalized in Table 1 with a stated purpose, scope, membership, decision criteria, and an explicit acknowledgment of open questions (e.g., who adjudicates “justification” for a post-gastrulation model). They are not claimed to be adopted, endorsed, or implemented by any body, and are presented as a starting point for policy discussion rather than as established governance.

## 3. Methods

This is a conceptual and mathematical modeling study. No human participants, animal subjects, identifiable genomic data, or biological samples were used at any stage. All reported scores are outputs of a deterministic formula and, where stated, of a stochastic simulation built on that formula; no data were collected, and no inferential hypothesis tests were performed, consistent with the exploratory, non-empirical nature of the work.

### 3.1. Audit of the originally proposed formula

The submitted formula was GEAF = ((A x E) – (C x I))/ (P + R) x 100, where A is an accessibility percentage (0–100), E an ethical compliance score (0–1), C a treatment cost in thousands of US dollars, I a Gini-like inequality coefficient (0–1), P a policy-strength scale (1–10), and R a research-efficiency factor (0–1). We evaluated this formula against four criteria, following standard practice in multi-criteria index construction [18,19]:

(a) Dimensional consistency. A x E produces a quantity on a 0–100 scale; C x I produces a quantity on a scale of roughly 0 to several thousand (since C is expressed in thousands of dollars). Subtracting the second from the first is dimensionally improper: the difference is dominated almost entirely by C x I regardless of the values of A and E, so the formula does not actually weigh accessibility and ethics against cost and inequality in any interpretable way – it is numerically swamped by cost.
(b) Boundedness and interpretability. Because the numerator is unbounded below (C can be arbitrarily large) and the denominator can be small (P + R can approach 1), the output has no defined minimum, maximum, or zero-point interpretation. The original formulation reported scores from −400 to +745 without specifying what any particular value means.
(c) Denominator behavior. Placing (P + R) in the denominator means that, holding the numerator fixed, increasing research efficiency or policy strength always increases the magnitude of the score, including in cases where the numerator is negative, where an increase in the denominator drives the (negative) score toward zero rather than in a direction that is independently interpretable as “better.” This produces behavior that is not clearly wrong, but that was not derived or justified theoretically in the original formulation.
(d) Scenario-formula consistency. The seven illustrative scenarios in the original Table 1 used cost inputs of $50,000 to $500,000, roughly an order of magnitude below verified real-world gene-therapy list prices, which as of this writing range from approximately $2.2 million (Casgevy, Vertex [2] to $4.25 million (Lenmeldy, Orchard [3]. This internal inconsistency, not previously flagged, means the original scenarios did not actually represent the cost environment the paper otherwise describes.

On the basis of (a)-(d), we concluded that the original formula could not be defended by re-deriving weights or re-normalizing in place; it required reconstruction rather than repair.

### 3.2. Reconstructed formula (GEAF’)

We reconstructed the index using multi-attribute utility theory [18], the same general framework invoked, but not actually implemented, in the original formulation. Each of the six input variables is first transformed into a dimensionless, direction-consistent component on the interval [0,1], where 1 always represents the more favorable state for equity and access. The transformed components are then combined as a weighted arithmetic mean and rescaled to a 0–100 index:


$$\begin{array}{c} \text{A}\_\text{n }=\text{ A}/\text{ 100 E}\_\text{n }=\text{ E C}\_\text{n }=\;\text{1}\;-\text{ min}(\text{C}/\text{ Cmax},\;\text{1})\text{ I}\_\text{n }=\;\text{1}\;-\text{ I P}\_\text{n }=\text{ P}/\text{ 10 R}\_\text{n }=\text{ R} \end{array}$$

$$\begin{array}{c} \text{GEA}{\text{F}}^{\prime }\;=\text{ 100 x }[\text{wA}\cdot \text{A}\_\text{n }+\text{ wE}\cdot \text{E}\_\text{n }+\text{ wC}\cdot \text{C}\_\text{n }+\text{ wI}\cdot \text{I}\_\text{n }+\text{ wP}\cdot \text{P}\_\text{n }+\text{ wR}\cdot \text{R}\_\text{n}\},\;\Sigma \text{w }=\;\text{1} \end{array}$$

C_max is a modeling normalization ceiling, not an affordability estimate or a claim about typical treatment cost: it fixes the point at which the affordability component (C_n) reaches its floor of 0. In this manuscript we set C_max = $4.25 million (4,250 in thousands), corresponding to the verified US wholesale acquisition cost of Lenmeldy (atidarsagene autotemcel, Orchard [3], the highest publicly reported list price for an approved gene therapy at the time of writing (Orchard [3]. We emphasize that this anchor was selected only to fix the normalization scale, and Table 8 reports that GEAF’ outputs are sensitive to this specific choice of ceiling. In the absence of a validated empirical weighting scheme (e.g., from a discrete-choice experiment or multi-criteria decision analysis with stakeholders), we use equal weights (w = 1/6 for each component) as a transparent, neutral default and treat unequal weighting as an explicit avenue for future empirical work rather than as a hidden assumption; equal weighting is reported, not claimed to be uniquely correct.

This reconstruction satisfies four validity requirements for a composite index of this kind: all six components are now on the same [0,1] scale before combination (resolving dimensional incompatibility); the index is bounded on [0,100] by construction (resolving the interpretability concern); the arithmetic-mean structure has no denominator-driven distortion (resolving the P + R behavior concern); and Section 3.4 re-specifies scenario cost inputs to be consistent with the cost literature cited elsewhere in the manuscript (resolving the scenario-formula inconsistency).

### 3.3. Parameter definitions, data sources, and level of evidence

Table 2 summarizes the definition, scale, source/derivation, and evidentiary status of each parameter.

**Table 2:** GEAF’ parameter definitions and evidentiary status.

Parameter	Scale	Definition	Source/ derivation	Evidence level
A – Accessibility Index	0-100 (%)	Estimated share of the target population with realistic access to the therapy	Illustrative assumption in this paper; empirically measurable from health-system coverage statistics [20] in a calibrated application	Assumption (this paper); Empirically estimable (future work)
E – Ethical Compliance Score	0-1	Composite adherence to ISSCR-type governance guidelines and trial inclusivity norms	Illustrative assumption; would require a defined scoring rubric against [8,16] provisions to be measured, not assumed	Assumption (this paper); requires rubric development
C – Cost per treatment	US$, thousands	List or net price per treatment course	Anchored to verified published list prices (Vertex [2] [Casgevy, $2.2M]; CSL [5] [Hemgenix, $3.5M]; bluebird bio, [4] [Lyfgenia, $3.1M]; Orchard [3] [Lenmeldy, $4.25M]) for the baseline/no-intervention scenario and the normalization ceiling; reduced-cost scenarios are illustrative percentage reductions, not published post-intervention prices	Published estimate (baseline anchor and C_max); Assumption (reduced-cost scenarios)
I – Inequality Factor	0-1 (Gini-like)	Distributional gap in access across sub-populations	Illustrative assumption; a calibrated application would compute this from a concentration index on real coverage data [21]	Assumption (this paper); empirically estimable (future work)
P – Policy Intervention Strength	1-10 (ordinal)	Composite rating of funding, subsidy, and regulatory support	Illustrative assumption; not derived from a validated policy-scoring instrument	Assumption
R – Research/System Efficiency	0-1	Composite rating of manufacturing, R&D, and delivery-system efficiency gains	Illustrative assumption	Assumption

Consistent with data-transparency expectations, every scenario value used below is labeled as either a “published estimate” (with a citation supporting the specific number) or an “illustrative assumption” (a value chosen for algebraic demonstration, not asserted to be measured). No value in this manuscript is described as empirically validated unless a specific citation supports that exact figure.

### 3.4. Scenario construction

We reconstructed the seven illustrative scenarios so that the baseline (no-intervention) cost value is anchored to a verified published gene-therapy list price, and so that reduced-cost scenarios are labeled as illustrative percentage reductions from that baseline rather than as independently sourced prices (no published, generalizable post-intervention price dataset for tiered gene-therapy pricing currently exists, a data gap we return to in Limitations). Table 3 gives the full input set and the justification/evidence label for each scenario; all seven are illustrative policy scenarios constructed for algebraic demonstration and are not empirical observations, forecasts, or validated projections.

**Table 3:** Reconstructed illustrative scenarios: Inputs and justification.

Scenario	A	E	C ($‘000)	I	P	R	Justification/ evidence label
S1 Baseline (no equity intervention)	20	0.6	3500	0.7	2	0.2	C = $3.5M is an illustrative baseline anchored to the verified published US list price of Hemgenix [(etranacogene dezaparvovec, CSL 5], not a computed average of a price range. A, E, I, P, R = illustrative assumptions consistent with the general LMIC coverage narrative in [20].
S2 Tiered pricing (LMIC discount, moderate)	55	0.8	1750	0.45	6	0.4	C = 50% reduction from S1, consistent with vaccine-style tiered pricing precedent [22]. Not an empirical projection for gene therapy.
S3 Tiered pricing + subsidy (optimized LMIC)	75	0.9	700	0.25	8	0.6	C = 80% reduction from S1. Illustrative upper bound; no empirical LMIC gene-therapy pricing dataset currently exists to calibrate this value precisely.
S4 LMIC infrastructure-constrained scenario	30	0.65	3000	0.8	3	0.2	Reflects [23] finding that LMIC genomic infrastructure and trial representation remain limited; C only modestly reduced due to supply-chain/cold-chain constraints [23].
S5 Public-private partnership scenario	65	0.85	1200	0.35	7	0.55	Illustrative; loosely modelled on CMS Cell and Gene Therapy Access Model outcome-based payment logic [24].
S6 High-income, no equity policy	60	0.8	2800	0.5	4	0.4	High nominal accessibility but no equity-specific policy lever; C close to list price.
S7 High-income, optimized equity policy	90	0.95	900	0.15	9	0.8	Best-case, fully optimized illustrative scenario combining tiered pricing, AI-driven manufacturing cost reduction, and strong policy/research investment.

### 3.5. Sensitivity and uncertainty analysis

Four complementary analyses were conducted to characterize model sensitivity and uncertainty, extending beyond a single one-way cost variation:

One-way sensitivity: each parameter was varied + −20% from its baseline (Scenario 1) value while holding all others fixed, and the resulting change in GEAF’ was recorded and ranked (tornado analysis).

Two-way sensitivity: GEAF’ was recomputed across a full grid of (i) cost (C) x inequality (I), and (ii) accessibility (A) x cost (C), with remaining parameters fixed at Scenario 1 values, to visualize interaction effects.

Edge-case testing: GEAF’ was evaluated at the algebraic extremes of every parameter (all values simultaneously best-case; all values simultaneously worst-case; cost at and beyond the normalization ceiling) to confirm boundedness and monotonicity before any substantive interpretation was attempted.

Probabilistic sensitivity analysis (Monte Carlo): 10,000 iterations were drawn for two parameter-uncertainty regimes – a “baseline/no-intervention” regime and an “equity-intervention” regime – using Beta distributions for the [0,1]-bounded parameters (A/100, E, I, R) and a log-normal distribution for cost, with distributional means and spreads chosen to be broadly consistent with the corresponding scenario in Table 3. These distributions are explicitly stated modeling choices intended to characterize plausible uncertainty around illustrative assumptions; they are not derived from an empirical dataset of parameter variability, and are labeled as such in Results and Limitations. Table 4 documents the exact distributional form and parameters used for each regime, so that the probabilistic analysis is fully reproducible and its assumption-based (not empirical) status is explicit.

**Table 4:** Monte Carlo distributional assumptions by parameter and uncertainty regime (10,000 iterations each; seed = 20260907).

Parameter	Regime	Distribution	Central tendency	Evidentiary status
Accessibility, A/100	Baseline	Beta(2,6)	Mean ≈ 0.25 (i.e., A ≈ 25)	Modeling assumption, not empirical
Accessibility, A/100	Equity-intervention	Beta(6,3)	Mean ≈ 0.67 (i.e., A ≈ 67)	Modeling assumption, not empirical
Ethical compliance, E	Baseline	Beta(6,4)	Mean ≈ 0.60	Modeling assumption, not empirical
Ethical compliance, E	Equity-intervention	Beta(9,2)	Mean ≈ 0.82	Modeling assumption, not empirical
Cost, C ($‘000)	Baseline	Log-normal(μ = ln(3200), σ = 0.35), truncated to [100, 6000]	Median ≈ 3,200	Modeling assumption anchored to baseline scenario order of magnitude, not an empirical price-variability estimate
Cost, C ($‘000)	Equity-intervention	Log-normal(μ = ln(1000), σ = 0.40), truncated to [50, 4250]	Median ≈ 1,000	Modeling assumption, not empirical
Inequality, I	Baseline	Beta(6,3)	Mean ≈ 0.67	Modeling assumption, not empirical
Inequality, I	Equity-intervention	Beta(2,6)	Mean ≈ 0.25	Modeling assumption, not empirical
Policy strength, P	Baseline	Triangular(min = 1, mode = 2, max = 5), rounded	Mode = 2	Modeling assumption, not empirical
Policy strength, P	Equity-intervention	Triangular(min = 4, mode = 8, max = 10), rounded	Mode = 8	Modeling assumption, not empirical
Research efficiency, R	Baseline	Beta(2,6)	Mean ≈ 0.25	Modeling assumption, not empirical
Research efficiency, R	Equity-intervention	Beta(5,3)	Mean ≈ 0.63	Modeling assumption, not empirical

All entries in Table 4 are stated, author-selected modeling assumptions used to characterize plausible uncertainty for illustrative purposes; none are derived from an empirical study of real-world parameter variability. The full generating code is in S1 Appendix.

All calculations were performed in Python 3.12 with NumPy 2.4; the complete, reproducible analysis code (model definition, sensitivity analysis, and Monte Carlo simulation) is provided in S1 Appendix (Supplementary Information) so that any reader can reproduce every number reported in this manuscript exactly. A 90% interval is reported throughout as the 5th-to-95th percentile range of the simulated distribution, which is distinct from the 10th-to-90th percentile range (an 80% interval).

### 3.6. Ethics approval and data governance

This study did not involve human participants, animal subjects, identifiable human genomic data, or biological material of any kind; accordingly, no ethics committee approval was required or sought. Because the paper discusses hypothetical future applications involving genomic data sharing (e.g., a proposed blockchain-enabled Global Genomic Commons), we note explicitly that these are prospective design proposals, not systems implemented or tested in this study, and that any future implementation would need to satisfy data-protection, informed-consent, community-governance, and benefit-sharing standards [e.g., [25–28] before deployment. No such implementation was undertaken here.

### 3.7. Data availability

This study did not generate or analyze primary datasets. All parameter values, scenario inputs, and analysis code are provided in full in the manuscript and its Supplementary Information (S1 Appendix), consistent with the Data Availability Statement: “All data are in the manuscript and/or supporting information files.”

## 4. Results

### 4.1. Edge-case behavior

At the algebraic worst case (A = 0, E = 0, C = C_max, I = 1, P = 0, R = 0), GEAF’ = 0.0. At the algebraic best case (A = 100, E = 1, C = 0, I = 0, P = 10, R = 1), GEAF’ = 100.0. Cost values at or beyond C_max produce an identical (floored) affordability component, preventing the unbounded behavior of the original formulation. GEAF’ is monotonically increasing in A, E, P, and R, and monotonically decreasing in C and I, confirmed numerically across the full input range (S1 Appendix). This establishes the index’s output range and directionality, neither of which was defined in the original formulation.

### 4.2. Scenario analysis

Table 5 and Fig 1 report GEAF’ for the seven reconstructed scenarios described in Table 3. The no-intervention baseline (S1), anchored to the verified Hemgenix list price [($3.5 million; CSL [5]] as an illustrative starting point, yields GEAF’ = 27.9, reflecting low accessibility and high cost/inequality. Progressive tiered-pricing and subsidy scenarios (S2, S3) raise the score to 58.1 and 77.2 respectively. The infrastructure-constrained LMIC scenario (S4) remains low (32.4) despite a moderate cost reduction, because accessibility and inequality – not cost alone – continue to constrain the index, consistent with [23] findings on LMIC genomic infrastructure gaps. The fully optimized high-income scenario (S7) reaches 86.5, the ceiling case among the illustrative scenarios.

**Table 5:** GEAF’ scores for the seven reconstructed scenarios.

Scenario	A	E	C	I	P	R	GEAF’
S1	20	0.6	3500	0.7	2	0.2	27.94
S2	55	0.8	1750	0.45	6	0.4	58.14
S3	75	0.9	700	0.25	8	0.6	77.25
S4	30	0.65	3000	0.8	3	0.2	32.4
S5	65	0.85	1200	0.35	7	0.55	68.63
S6	60	0.8	2800	0.5	4	0.4	50.69
S7	90	0.95	900	0.15	9	0.8	86.47

**Fig 1:**
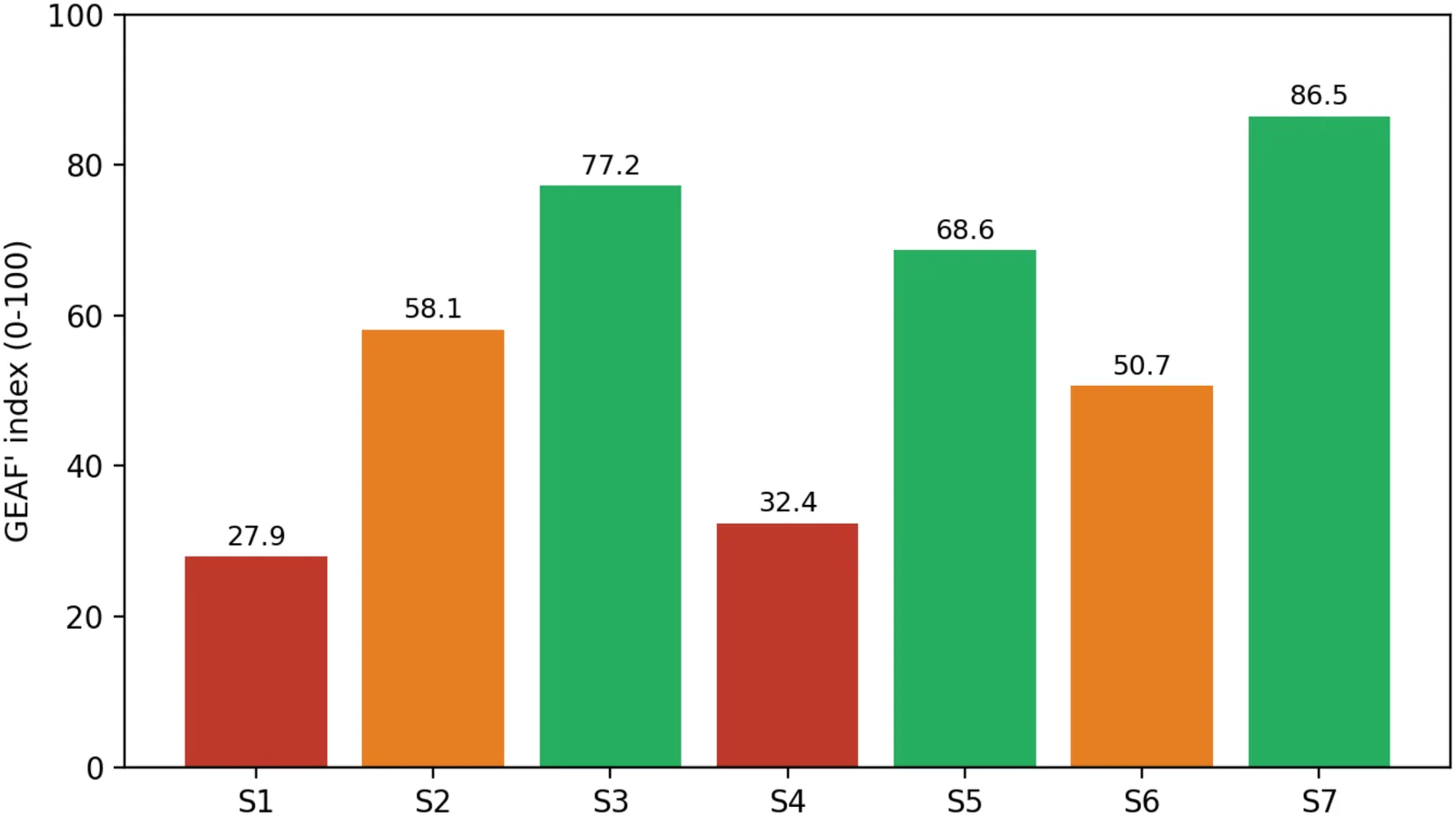
Reconstructed GEAF’ scores across seven illustrative scenarios (0-100 scale; color indicates low/moderate/high band for visual reference only, not a validated clinical or policy threshold).

We deliberately avoid asserting a specific cost-reduction percentage (e.g., an unsupported “up to 70%” figure proposed in an earlier version of this formulation) as a general finding. The cost reductions embedded in scenarios S2-S3, S5, and S7 are stated modeling assumptions used to illustrate how GEAF’ responds to policy-relevant inputs, not empirical projections of what any real policy will achieve. Any real-world cost-reduction claim would need to be derived from an economic model specific to a named therapy, market, and policy instrument, which is beyond the scope of this conceptual paper and is flagged in Section 5.5 (Limitations) as a priority for future empirical work.

### 4.3. One-way (tornado) sensitivity analysis

Table 6 and Fig 2 report one-way sensitivity results for the baseline scenario (S1), varying each parameter ±20% while holding the others fixed. Cost (C) produced the largest swing in GEAF’ (5.5 points), followed by the inequality factor (I, 4.7 points) and ethical compliance (E, 4.0 points); accessibility (A), policy strength (P), and research efficiency (R) produced smaller, equal-magnitude swings (1.3 points each) because of the symmetric equal-weighting scheme used here. This ranking is consistent with the qualitative claim in the original formulation that cost and inequality are the dominant equity barriers, but is now derived from a dimensionally coherent model rather than from an admittedly ad hoc formula.

**Table 6:** One-way sensitivity analysis results, baseline scenario (S1), ± 20% variation.

Parameter	GEAF’ (low input)	GEAF’ (high input)	Absolute swing
Cost (C)	30.7	25.2	5.5
Inequality (I)	30.3	25.6	4.7
Ethical compliance (E)	25.9	29.9	4.0
Accessibility (A)	27.3	28.6	1.3
Policy strength (P)	27.3	28.6	1.3
Research efficiency (R)	27.3	28.6	1.3

**Fig 2:**
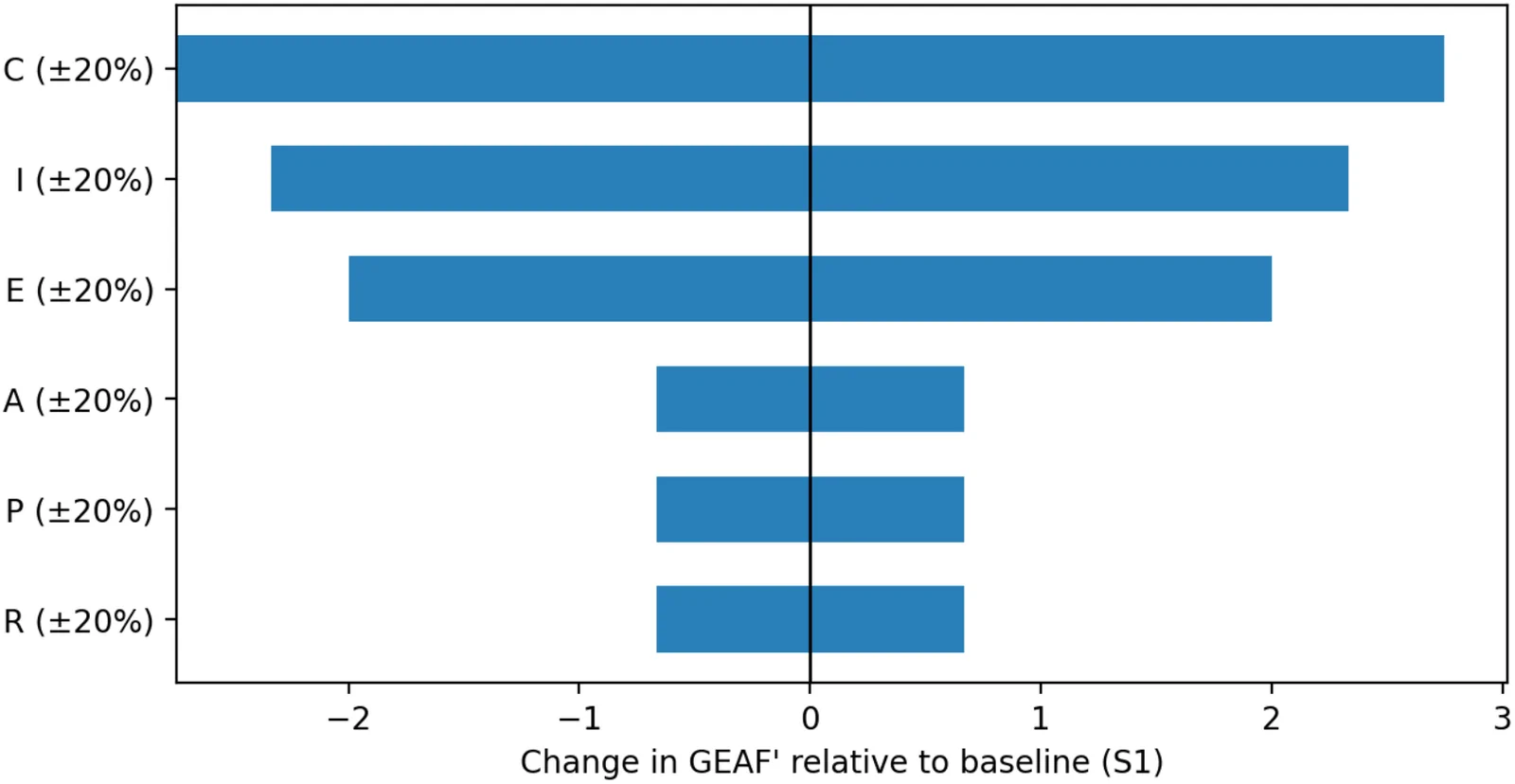
One-way (tornado) sensitivity analysis, baseline scenario (S1).

### 4.4. Two-way sensitivity analysis

Fig 3 shows GEAF’ as a joint function of cost (C) and the inequality factor (I), with accessibility, ethical compliance, policy strength, and research efficiency fixed at Scenario 1 values. The two parameters combine additively under the reconstructed formula (no interaction term is included in the current specification), producing smooth, monotonic contours rather than the abrupt sign changes possible under the original formula’s division structure. Fig 4 shows the corresponding accessibility (A) x cost (C) surface.

**Fig 3:**
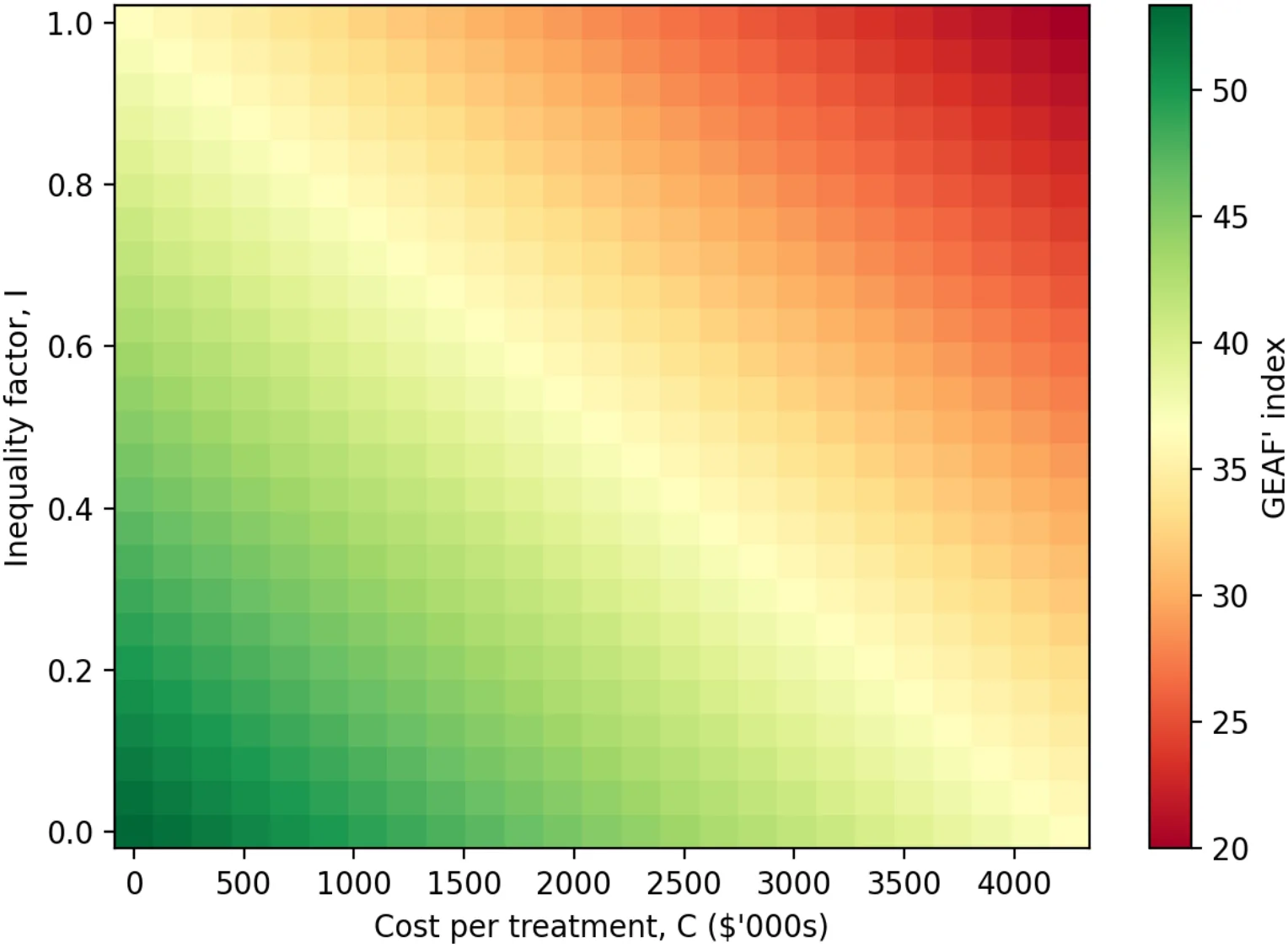
Two-way sensitivity: cost (C) x inequality (I); other parameters fixed at Scenario 1 values.

**Fig 4:**
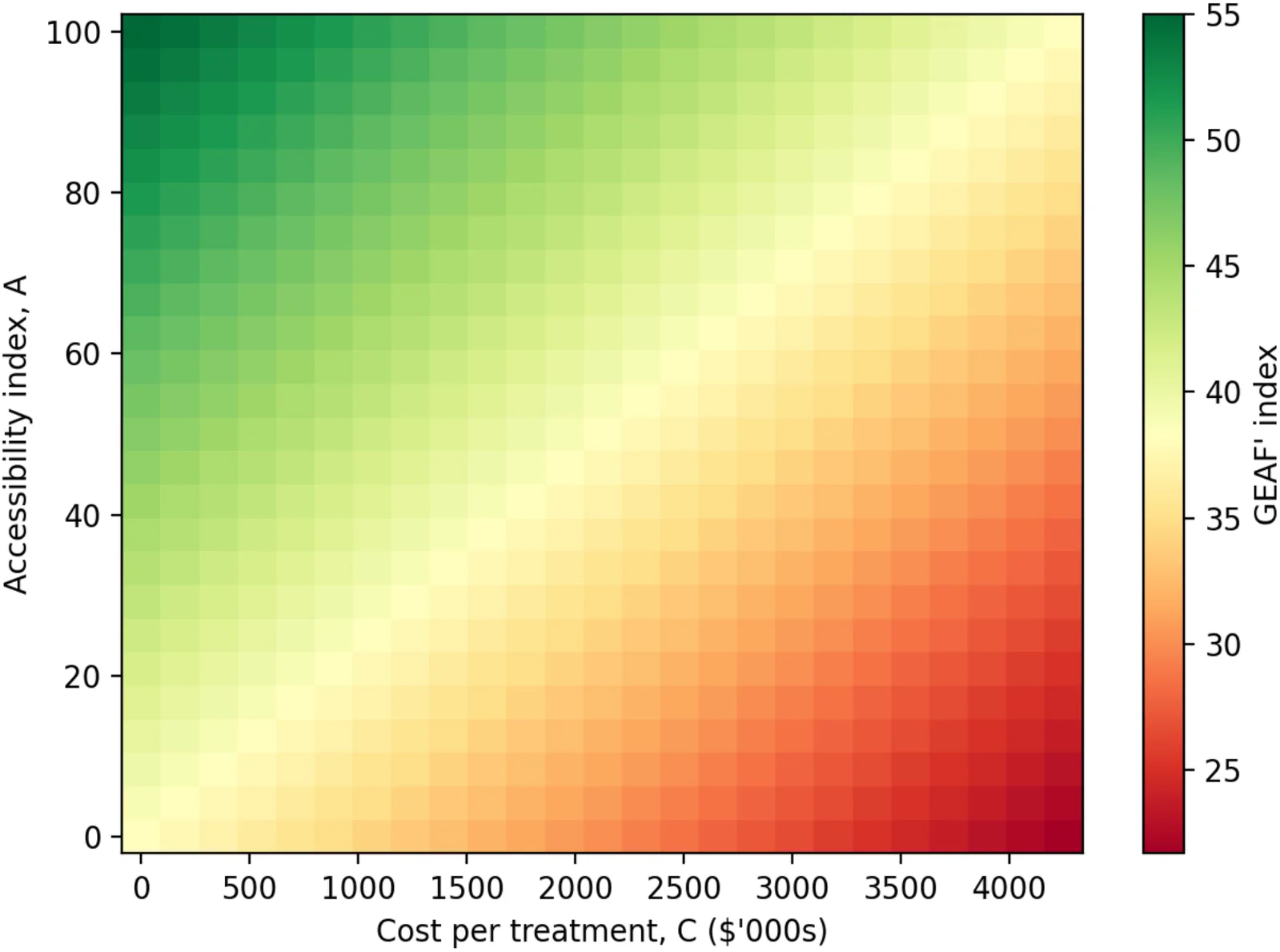
Two-way sensitivity: accessibility (A) x cost (C); other parameters fixed at Scenario 1 values.

Because GEAF’ is constructed as an equally weighted arithmetic mean of normalized components, the aggregation is compensatory by design: with equal weights, a given increase in one normalized component (for example, C_n) can exactly offset an equal-sized decrease in another (for example, A_n), one-for-one, since each contributes the same weight (1/6) to the total. This is a mathematically accurate and expected property of an arithmetic-mean composite index, not a defect, but it has a substantive implication worth stating plainly: under the current equal-weighted specification, sufficiently large affordability gains can numerically offset very low accessibility in the index (and vice versa), because the aggregation does not penalize an extreme low value in one component beyond its fixed 1/6 weight. Whether cost reduction should be treated as fully able to substitute for an accessibility shortfall (or an ethical-compliance shortfall) in this way is a normative and policy question, not a mathematical one; the current version of GEAF’ does not resolve it, and Table 8 lists this compensatory-versus-non-compensatory aggregation choice as an explicit limitation and an avenue for future work (e.g., a minimum-component or multiplicative floor could be introduced if non-compensatory behavior is judged more appropriate for a specific policy application).

### 4.5. Probabilistic sensitivity analysis (Monte Carlo)

Table 4 (Methods) documents the distributions used. Under the baseline/no-intervention uncertainty regime (10,000 iterations, seed = 20260907), GEAF’ had a mean of 32.6 (median 32.5; 90% interval, 5th-95th percentile, approximately 22.9-42.9). Under the equity-intervention uncertainty regime, GEAF’ had a mean of 72.4 (median 72.6; 90% interval approximately 62.9-81.1). The two distributions did not overlap within the simulated parameter ranges (Fig 5), indicating that the qualitative separation between “no-intervention” and “equity-intervention” scenarios is robust to the specific input distributions used here, though the numerical intervals themselves depend on the stated, non-empirical distributional assumptions in Table 4 and should not be read as confidence intervals around a measured real-world quantity. These exact figures are reproduced by the code in S1 Appendix.

**Fig 5:**
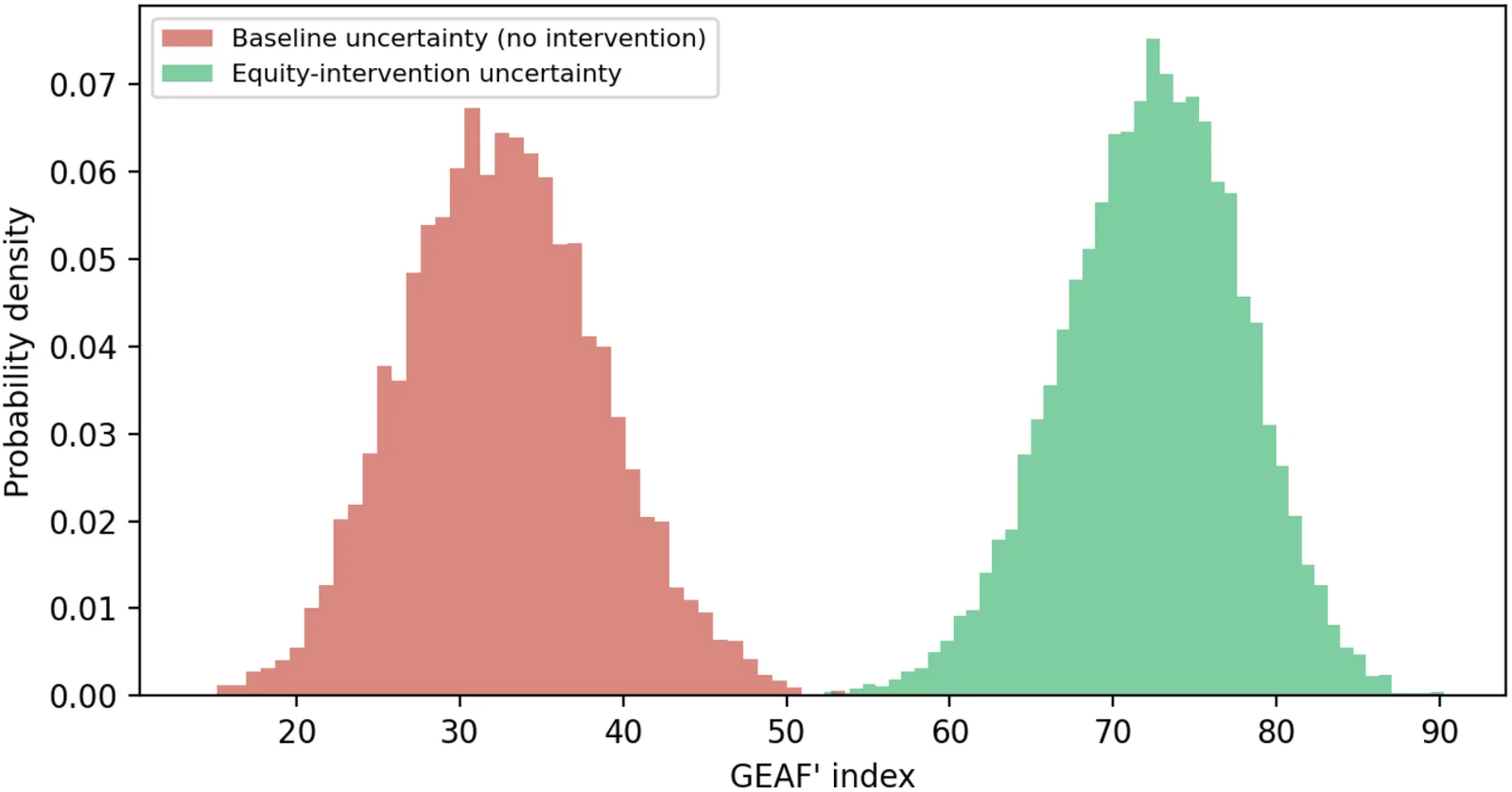
Monte Carlo probabilistic sensitivity analysis (10,000 iterations per uncertainty regime).

### 4.6. Comparison with existing frameworks

Table 7 positions GEAF’ conceptually against established cost-effectiveness and equity-weighting frameworks. We did not attempt a direct empirical GEAF’-versus-ICER comparison on a shared real-world dataset, because no publicly available dataset was identified that reports accessibility, ethical-compliance, inequality, policy-strength, and research-efficiency parameters alongside a conventional ICER for the same therapy-jurisdiction pair; fabricating such a comparison would misrepresent the state of the evidence. This is stated as an explicit limitation rather than presented as a completed analysis [30].

**Table 7:** Conceptual comparison of GEAF’ with existing cost-effectiveness and equity frameworks.

Framework	Primary objective	Cost	Accessibility	Distributional inequality	Ethics	Policy strength	Strengths	Limitations	Relationship to GEAF’
ICER (incremental cost-effectiveness ratio)	Value-for-money per unit of health gain	Yes	No (implicitly, via willingness-to-pay threshold)	No	No	No	Widely used, well understood by payers	Ignores distribution of benefit; single-dimensional	GEAF’ adds explicit equity/ethics/access terms ICER omits
QALY/DALY-based HTA	Health-gain quantification	Indirect	No	No	No	No	Standardized outcome unit	Does not capture access or ethical-compliance dimensions	Complementary; GEAF’ does not replace QALY/DALY measurement
Distributional cost-effectiveness analysis (DCEA)	Equity-weighted cost-effectiveness	Yes	Partial	Yes	No	No	Formally incorporates equity weights [29]	Requires detailed subgroup data; complex to implement	Conceptually closest existing framework; GEAF’ is a simpler composite alternative, not a substitute for a full DCEA
Extended cost-effectiveness analysis (ECEA)	Adds financial-risk-protection dimension to CEA	Yes	Partial	Yes	No	No	Captures catastrophic health expenditure risk	Data-intensive; not genomics-specific	Complementary equity lens; not incorporated in current GEAF’ version
WHO health equity assessment frameworks	Population-level equity monitoring	No (typically)	Yes	Yes	No	No	Policy-relevant, internationally recognized	Not a single quantitative composite index	GEAF’ operationalizes some WHO equity concepts as a scenario-comparison index
GEAF’ (this paper)	Composite policy-scenario index for genomic/regenerative therapies	Yes	Yes	Yes	Yes	Yes	Transparent, bounded, reproducible, explicit ethics/policy terms	Not yet empirically calibrated or validated; equal-weighting is a stated assumption	–

## 5. Discussion

### 5.1. Principal findings

Reconstructing GEAF as a normalized, equally weighted composite of six [0,1] components resolves specific mathematical defects in the original formulation: the index is now dimensionally consistent, bounded on [0,100], and monotonic in every input, and its behavior under one-way, two-way, and probabilistic sensitivity analysis is well defined and reproducible. Across illustrative scenarios anchored to cited real-world price literature, GEAF’ separates a no-intervention baseline (27.9) from a fully optimized equity-policy scenario (86.5), with cost and distributional inequality identified as the two largest single-parameter drivers under baseline conditions – a finding consistent with, but now derived independently of, the qualitative narrative in the original formulation about cost and inequality as the primary equity barriers.

### 5.2. Relationship to existing frameworks

GEAF’ is best understood as a lightweight, transparent complement to – not a replacement for – established health-technology-assessment methods such as ICER-based evaluation and distributional cost-effectiveness analysis (DCEA) [31,32]. Its comparative advantage is combining accessibility, ethical-compliance, and policy/research-capacity terms that conventional CEA/ICER frameworks do not represent, in a form simple enough to compute without specialized software; its comparative disadvantage is that, unlike DCEA, it does not (in its current form) model utility curves, opportunity cost, or budget constraints explicitly. We do not claim GEAF’ outperforms DCEA or ICER-based analysis; Table 7 positions it as occupying a different, complementary niche focused on rapid, transparent scenario comparison rather than formal resource-allocation optimization.

### 5.3. Policy and ethical implications

If further developed and empirically calibrated, an index of this kind could give policymakers a transparent way to compare how alternative pricing, subsidy, or regulatory instruments are jointly expected to affect accessibility, cost burden, distributional equity, and stated ethical-compliance goals for a specific therapy in a specific setting. We emphasize “if further developed and empirically calibrated”: in its current, illustrative-scenario form, GEAF’ is a demonstration of a mathematically coherent structure, not a validated decision tool, and should not be used to justify a specific pricing or reimbursement decision without prior empirical calibration of A, E, I, P, and R against real data for the therapy and jurisdiction in question. The ethical-compliance component (E) in particular reduces a genuinely contested area of moral philosophy (procreative beneficence versus deontological objections to enhancement; contested views on embryo moral status) to a single number; we treat this explicitly as a structured normative simplification useful for scenario comparison, not as a claim that ethical reasoning can be fully captured numerically.

### 5.4. Strengths

The principal strengths of GEAF’ as reconstructed here are: (i) full reproducibility, since every number in this manuscript is generated by openly provided code rather than manual arithmetic; (ii) explicit separation of published-estimate parameters from illustrative-assumption parameters in every table; (iii) systematic sensitivity and Monte Carlo analysis rather than a single + −20% cost check; and (iv) removal of quantitative claims (e.g., an earlier proposed 70% cost-reduction figure and projected 2035 global averages) that could not be traced to a specific supporting derivation.

### 5.5. Limitations

## 6. Conclusion

This paper reconstructs the Genomic Equity Accessibility Framework from a dimensionally inconsistent, unbounded expression into a normalized, bounded, monotonic composite index (GEAF’) built on multi-attribute utility theory, and subjects it to edge-case, one-way, two-way, and Monte Carlo sensitivity testing. Applied to seven illustrative scenarios whose cost inputs are anchored to real-world price literature, GEAF’ distinguishes clearly between low-equity and high-equity policy configurations in a way that is transparent and reproducible. This is presented as a methodological and conceptual contribution: GEAF’ has not been empirically calibrated or validated against real-world outcome data, unsupported quantitative claims (including an earlier proposed 70% cost-reduction figure and long-range projections) have been removed rather than retained without support, and a specific empirical validation agenda is set out in Table 8 as the necessary next step before GEAF’ could inform actual health-technology-assessment or resource-allocation decisions.

**Table 8:** Limitations, their significance, and future mitigation.

Limitation	Significance for interpretation	Proposed mitigation/ future work
No empirical calibration of A, E, I, P, or R against real accessibility, ethics-compliance, or inequality data for any named therapy/jurisdiction	High – scores should be read as illustrative, not predictive, until calibrated	Prospective calibration study using WHO/HTA-agency coverage data, a defined ISSCR-based ethics-compliance rubric, and country-level concentration indices
Equal weighting (1/6 each) is a modeling default, not an empirically or normatively derived weighting scheme	Moderate – alternative weights would shift relative scenario rankings, though not the qualitative direction of each parameter’s effect	Multi-criteria decision analysis or discrete-choice experiment with patients, clinicians, and payers to derive empirically grounded weights
Cost normalization ceiling (C_max = $4.25M, anchored to Lenmeldy’s verified list price) fixes the affordability scale but is a modeling choice, not an empirical estimate of a typical or maximum sustainable price	Moderate – shifts the absolute (not relative) scale of the affordability component; a different verified anchor (e.g., a lower- or higher-priced approved therapy) would shift all GEAF’ scores in this manuscript	Sensitivity analysis across alternative C_max values; empirical price-distribution data across approved gene therapies
The equal-weighted arithmetic-mean aggregation is compensatory by construction: a sufficiently large gain in one normalized component can numerically offset a large deficit in another (Section 4.4), which may not always be normatively desirable (e.g., very low accessibility offset by high affordability)	Moderate – affects interpretation of scenarios with an extreme value in any single component; does not affect the correctness of the reported scores	Evaluate non-compensatory or partially compensatory alternative aggregation rules (e.g., minimum-component floors, multiplicative terms) once stakeholder input on acceptable trade-offs is available
No direct empirical comparison against ICER on a shared dataset was performed	Moderate – Table 7’s comparison is conceptual, not empirically demonstrated	Identify or construct a dataset reporting both ICER and GEAF’ components for the same therapy-jurisdiction pairs
Scenario cost-reduction magnitudes (e.g., 50%, 80%) are illustrative assumptions, not derived from a specific economic model of any named policy instrument	Moderate – Results should not be read as forecasts of achievable cost reduction	Therapy- and market-specific economic modeling of tiered pricing, patent pooling, or subsidy mechanisms
The model currently combines components additively with no interaction terms; real equity dynamics may involve threshold or interaction effects (e.g., cost reduction below a threshold suddenly unlocking accessibility)	Low-to-moderate - may understate some real-world nonlinearities	Explore alternative aggregation functions (e.g., multiplicative or threshold terms) once empirical data are available to justify them
The ethical-compliance (E) component numerically represents a domain of contested moral philosophy	Conceptual – inherent to any attempt to quantify ethics	Explicit qualitative discussion (Section 5.3); E should be interpreted as a structured compliance/process indicator, not a moral verdict

## Supporting information

S1 Appendix Reproducible Analysis Code. Complete analysis code used to generate the GEAF′ calculations, scenario results, sensitivity analyses, and Monte Carlo simulations reported in the manuscript.
(DOCX)
