# Supplementary material for: The Genomic Equity Accessibility Framework (GEAF): A dimensionally consistent framework for modeling affordability and equity in genomic and regenerative therapies
Source: PLoS One. 2026 Oct 6;21(10):e0359782. doi: 10.1371/journal.pone.0359782 (PMC13641711; doi:10.1371/journal.pone.0359782)
Supplement: Supplementary file 1 [file pone.0359782.s001.docx]

**S1 Appendix**

**Reproducible Analysis Code**

*The Python/NumPy code below reproduces every GEAF′ score, sensitivity result, and Monte Carlo statistic reported in the manuscript “The Genomic Equity Accessibility Framework (GEAF): A Dimensionally Consistent Framework for Modeling Affordability and Equity in Genomic and Regenerative Therapies” (PONE-D-26-32218R1) exactly, with the random seed fixed at 20260907.*

```
import numpy as np
import json

np.random.seed(20260907)

# ---------- Reconstructed GEAF' model ----------
# All raw inputs converted to normalized "goodness" components on [0,1],
# then combined as a weighted arithmetic mean, scaled to 0-100.
# C_MAX is a modeling normalization ceiling, anchored to the verified US
# wholesale acquisition cost of Lenmeldy (atidarsagene autotemcel, Orchard
# Therapeutics, FDA approval March 2024): C_MAX = 4250 (in $'000s).

C_MAX = 4250.0  # $'000s. Modeling normalization ceiling only (NOT an affordability estimate),
                 # anchored to the verified US wholesale acquisition cost of Lenmeldy
                 # (atidarsagene autotemcel, Orchard Therapeutics, FDA approval March 2024),
                 # $4.25 million -- the highest publicly reported list price for an approved
                 # gene therapy at the time of writing (Orchard Therapeutics, 2024).

def normalize(A, E, C, I, P, R, c_max=C_MAX):
    A_n = np.clip(A/100.0, 0, 1)
    E_n = np.clip(E, 0, 1)
    C_n = np.clip(1 - np.minimum(C, c_max)/c_max, 0, 1)   # affordability (higher=cheaper)
    I_n = np.clip(1 - I, 0, 1)                             # equity (higher = less inequality)
    P_n = np.clip(P/10.0, 0, 1)
    R_n = np.clip(R, 0, 1)
    return A_n, E_n, C_n, I_n, P_n, R_n

DEFAULT_WEIGHTS = dict(A=1/6, E=1/6, C=1/6, I=1/6, P=1/6, R=1/6)

def geaf_prime(A, E, C, I, P, R, weights=DEFAULT_WEIGHTS, c_max=C_MAX):
    A_n, E_n, C_n, I_n, P_n, R_n = normalize(A, E, C, I, P, R, c_max)
    score = (weights['A']*A_n + weights['E']*E_n + weights['C']*C_n +
             weights['I']*I_n + weights['P']*P_n + weights['R']*R_n)
    return 100*score

# ---------- Reconstructed illustrative scenarios ----------
# Cost values re-anchored to the price range the manuscript itself cites
# (Shah et al. 2023 $2-4M; bluebird bio 2025 $2-4.25M; Novartis 2025 $2.1M;
# Spark Therapeutics 2025 $850k/eye). All scenario rows are labelled
# ILLUSTRATIVE / ASSUMPTION unless a specific citation anchors the value.

scenarios = [
    dict(name="S1 Baseline (no equity intervention)",
         A=20, E=0.60, C=3500, I=0.70, P=2, R=0.20,
         note="C = $3.5M is an illustrative baseline anchored to the verified published US list price of Hemgenix (etranacogene dezaparvovec, CSL Behring, FDA approval 2022), not a computed average of a price range. A, E, I, P, R = illustrative assumptions consistent with the general LMIC coverage narrative in WHO (2022)."),
    dict(name="S2 Tiered pricing (LMIC discount, moderate)",
         A=55, E=0.80, C=1750, I=0.45, P=6, R=0.40,
         note="C = 50% reduction from S1, consistent with vaccine-style tiered pricing precedent (Kanavos et al. 2020). Not an empirical projection for gene therapy."),
    dict(name="S3 Tiered pricing + subsidy (optimized LMIC)",
         A=75, E=0.90, C=700, I=0.25, P=8, R=0.60,
         note="C = 80% reduction from S1. Illustrative upper bound; no empirical LMIC gene-therapy pricing dataset currently exists to calibrate this value precisely."),
    dict(name="S4 LMIC infrastructure-constrained scenario",
         A=30, E=0.65, C=3000, I=0.80, P=3, R=0.20,
         note="Reflects WHO (2025) finding that LMIC genomic infrastructure and trial representation remain limited; C only modestly reduced due to supply-chain/cold-chain constraints (World Health Organization, 2025)."),
    dict(name="S5 Public-private partnership scenario",
         A=65, E=0.85, C=1200, I=0.35, P=7, R=0.55,
         note="Illustrative; loosely modelled on CMS Cell and Gene Therapy Access Model outcome-based payment logic (CMS, 2024)."),
    dict(name="S6 High-income, no equity policy",
         A=60, E=0.80, C=2800, I=0.50, P=4, R=0.40,
         note="High nominal accessibility but no equity-specific policy lever; C close to list price."),
    dict(name="S7 High-income, optimized equity policy",
         A=90, E=0.95, C=900, I=0.15, P=9, R=0.80,
         note="Best-case, fully optimized illustrative scenario combining tiered pricing, AI-driven manufacturing cost reduction, and strong policy/research investment."),
]

rows = []
for s in scenarios:
    g = geaf_prime(s['A'], s['E'], s['C'], s['I'], s['P'], s['R'])
    A_n,E_n,C_n,I_n,P_n,R_n = normalize(s['A'], s['E'], s['C'], s['I'], s['P'], s['R'])
    rows.append(dict(scenario=s['name'], A=s['A'],E=s['E'],C=s['C'],I=s['I'],P=s['P'],R=s['R'],
                      A_n=round(A_n,3),E_n=round(E_n,3),C_n=round(C_n,3),I_n=round(I_n,3),P_n=round(P_n,3),R_n=round(R_n,3),
                      GEAF=round(g,2), note=s['note']))
    print(f"{s['name']:55s} GEAF'={g:6.2f}")

with open('scenarios_out.json','w') as f:
    json.dump(rows, f, indent=2)

print("\n--- Edge case checks ---")
print("Min all (worst case A=0,E=0,C=Cmax,I=1,P=0,R=0):", geaf_prime(0,0,C_MAX,1,0,0))
print("Max all (best case A=100,E=1,C=0,I=0,P=10,R=1):", geaf_prime(100,1,0,0,10,1))
print("Cost beyond cap (C=10000) same as C=Cmax:", geaf_prime(50,0.5,10000,0.5,5,0.5), "vs", geaf_prime(50,0.5,C_MAX,0.5,5,0.5))
print("Monotonic in A? A=10 vs A=90 (else baseline mid):",
      geaf_prime(10,0.5,2000,0.5,5,0.5), geaf_prime(90,0.5,2000,0.5,5,0.5))
print("Monotonic in I (higher inequality lowers score)? I=0.1 vs I=0.9:",
      geaf_prime(50,0.5,2000,0.1,5,0.5), geaf_prime(50,0.5,2000,0.9,5,0.5))
import numpy as np, json
from model import geaf_prime, C_MAX, normalize

np.random.seed(20260907)

# Baseline for one-way sensitivity = S1 midpoint values
base = dict(A=20, E=0.60, C=3500, I=0.70, P=2, R=0.20)

ranges = dict(
    A=(base['A']*0.8, base['A']*1.2),
    E=(max(0,base['E']*0.8), min(1,base['E']*1.2)),
    C=(base['C']*0.8, base['C']*1.2),
    I=(max(0,base['I']*0.8), min(1,base['I']*1.2)),
    P=(max(1,base['P']*0.8), min(10,base['P']*1.2)),
    R=(max(0,base['R']*0.8), min(1,base['R']*1.2)),
)

base_score = geaf_prime(**base)
tornado = []
for k,(lo,hi) in ranges.items():
    lo_params = dict(base); lo_params[k]=lo
    hi_params = dict(base); hi_params[k]=hi
    lo_s = geaf_prime(**lo_params)
    hi_s = geaf_prime(**hi_params)
    tornado.append((k, lo_s, hi_s, abs(hi_s-lo_s)))

tornado.sort(key=lambda x: -x[3])
print("Baseline GEAF' =", round(base_score,2))
print("One-way sensitivity (+-20%), sorted by swing:")
for k,lo_s,hi_s,swing in tornado:
    print(f"  {k}: low={lo_s:6.2f}  high={hi_s:6.2f}  swing={swing:6.2f}")

json.dump(dict(base=base_score, tornado=tornado), open('tornado_out.json','w'))

# Two-way sensitivity heatmap: C x I -> GEAF, others fixed at S1 baseline
C_grid = np.linspace(0, C_MAX, 25)
I_grid = np.linspace(0, 1, 25)
heat = np.zeros((len(I_grid), len(C_grid)))
for i, Ival in enumerate(I_grid):
    for j, Cval in enumerate(C_grid):
        heat[i,j] = geaf_prime(base['A'], base['E'], Cval, Ival, base['P'], base['R'])
np.savez('heatmap_CI.npz', C=C_grid, I=I_grid, Z=heat)

# Two-way: A x C -> GEAF
A_grid = np.linspace(0,100,25)
heat2 = np.zeros((len(A_grid), len(C_grid)))
for i, Aval in enumerate(A_grid):
    for j, Cval in enumerate(C_grid):
        heat2[i,j] = geaf_prime(Aval, base['E'], Cval, base['I'], base['P'], base['R'])
np.savez('heatmap_AC.npz', A=A_grid, C=C_grid, Z=heat2)

print("Heatmaps computed and saved.")
import numpy as np, json
from model import geaf_prime, C_MAX

np.random.seed(20260907)
N = 10000

def run_mc(mode="baseline"):
    if mode == "baseline":
        # centered near S1, moderate uncertainty
        A = np.clip(np.random.beta(2,6,N)*100, 0, 100)          # mean ~25
        E = np.clip(np.random.beta(6,4,N), 0, 1)                # mean ~0.6
        C = np.clip(np.random.lognormal(mean=np.log(3200), sigma=0.35, size=N), 100, 6000)
        I = np.clip(np.random.beta(6,3,N), 0, 1)                # mean ~0.67, skewed high
        P = np.clip(np.round(np.random.triangular(1,2,5,N)),1,10)
        R = np.clip(np.random.beta(2,6,N),0,1)
    else:  # optimized / equity-intervention uncertainty
        A = np.clip(np.random.beta(6,3,N)*100, 0, 100)          # mean ~67
        E = np.clip(np.random.beta(9,2,N), 0, 1)                # mean ~0.82
        C = np.clip(np.random.lognormal(mean=np.log(1000), sigma=0.4, size=N), 50, 4250)
        I = np.clip(np.random.beta(2,6,N), 0, 1)                # mean ~0.25
        P = np.clip(np.round(np.random.triangular(4,8,10,N)),1,10)
        R = np.clip(np.random.beta(5,3,N),0,1)
    scores = geaf_prime(A,E,C,I,P,R)
    return scores

base_scores = run_mc("baseline")
opt_scores = run_mc("optimized")

def summarize(x):
    return dict(mean=float(np.mean(x)), median=float(np.median(x)),
                p05=float(np.percentile(x,5)), p95=float(np.percentile(x,95)),
                p10=float(np.percentile(x,10)), p90=float(np.percentile(x,90)))

summary = dict(baseline=summarize(base_scores), optimized=summarize(opt_scores))
print(json.dumps(summary, indent=2))

pct_positive_gain = float(np.mean(opt_scores > base_scores.mean()))
print("P(optimized-uncertainty draw exceeds baseline mean):", pct_positive_gain)

np.savez('mc_out.npz', base=base_scores, opt=opt_scores)
json.dump(summary, open('mc_summary.json','w'), indent=2)
```
