# Supplementary material for: The Genomic Equity Accessibility Framework (GEAF): A dimensionally consistent framework for modeling affordability and equity in genomic and regenerative therapies
Source: PLoS One. 2026 Oct 6;21(10):e0359782. doi: 10.1371/journal.pone.0359782 (PMC13641711; doi:10.1371/journal.pone.0359782)
Supplement: Supplementary file 2 [file pone.0359782.s003.docx]

**Final Response to Reviewers**

*Manuscript: PONE-D-26-32218. Original title: “Revolutionizing the Genome: Ethical Innovations, Equitable Policies, and a Mathematical Framework for Affordable Regenerative Medicine.” Final title: “The Genomic Equity Accessibility Framework (GEAF): A Dimensionally Consistent Framework for Modeling Affordability and Equity in Genomic and Regenerative Therapies.”*

We thank the Academic Editor and both reviewers for a thorough, constructive review. The central and correct criticism - that the original GEAF formula was mathematically unsound and insufficiently justified - has been addressed by reconstructing the index from first principles (Methods, Model Reconstruction) rather than defending the original formulation. Each point below follows a consistent structure: the reviewer's comment, our response, the specific revision made, and its exact location in the final manuscript. We are explicit about the small number of requests we did not fulfill as literally worded (a direct empirical GEAF-vs-ICER comparison; Nigeria-specific epidemiological tables; three reviewer-suggested off-topic citations), and state why in each case.

# Response to the Academic Editor

***Please ensure the manuscript meets PLOS ONE style requirements, including file naming.***

**Response:** The manuscript follows PLOS ONE's structural conventions (title page, structured Abstract, standalone Methods before Results, Declarations) and this response follows PLOS ONE's file-naming convention.

**Specific Revision Made:** Full structural reorganization; consistent PLOS ONE file naming applied to all three final documents.

**Location in Revised Manuscript:** Whole-manuscript structure; filenames GEAF_PONE_D_26_32218_Final_Amended_Manuscript.docx, GEAF_PONE_D_26_32218_Original_to_Final_Track_Changes.docx, GEAF_PONE_D_26_32218_Final_Response_to_Reviewers.docx.

***Please provide a complete Data Availability Statement.***

**Response:** Added a complete Data Availability Statement appropriate to a conceptual/modeling study.

**Specific Revision Made:** “All data are in the manuscript and/or supporting information files”, with all parameters, scenarios, and analysis code disclosed in-text and in S1 Appendix.

**Location in Revised Manuscript:** Declarations – Data Availability Statement.

***Please update the submission to the PLOS LaTeX template.***

**Response:** Noted for final typesetting; the DOCX content is structured so that a PLOS LaTeX conversion at final submission is straightforward.

**Specific Revision Made:** No manuscript content change required; this is a production-format step to be completed at upload.

**Location in Revised Manuscript:** Not applicable to manuscript text.

***Please amend the author list so each author is linked to an affiliation.***

**Response:** The manuscript has a single author; affiliation is corrected to reflect only the institution that provided the research environment for this work.

**Specific Revision Made:** Affiliation reduced from eight listed institutional addresses to Strayer University alone; professional-society memberships and the author's own organizations (Eloi Holding, Inc.; David Oloche Foundation) are disclosed separately as memberships/funding/competing interests, not affiliations.

**Location in Revised Manuscript:** Title page (affiliation line); Acknowledgments; Funding Statement; Competing Interests.

***If reviewers recommend citing specific works, evaluate relevance; no obligation to include.***

**Response:** Each reviewer-suggested reference was evaluated individually and included only where substantively relevant.

**Specific Revision Made:** Three reviewer-suggested references (gut microbiota metabolic reprogramming; SUMOylation in oncology; functional foods/MAFLD) were evaluated and not included, as none bear on a genomic-therapy equity-index methods paper.

**Location in Revised Manuscript:** See Reviewer #2, Items 7-9 below; final Reference list.

# Response to Reviewer #1

***The GEAF formula lacks rigorous theoretical justification; the multiplicative/subtractive structure is arbitrary.***

**Response:** Agreed in full. The original formula was audited against four validity criteria (dimensional consistency, boundedness, denominator behavior, scenario-formula consistency) and found deficient on all four; it was reconstructed rather than defended.

**Specific Revision Made:** New Methods subsection auditing the original formula's defects, followed by a full reconstruction using multi-attribute utility theory: GEAF' = 100 × Σ(wᵢ × Xᵢ), six components normalized to [0,1], equal weights (1/6) as a stated default.

**Location in Revised Manuscript:** Methods, “Audit of the originally proposed formula” and “Reconstructed formula (GEAF′)” subsections.

***(a) Why multiply A×E rather than a weighted sum? Why subtract C×I rather than divide?***

**Response:** Resolved by reconstruction: a single, consistent aggregation rule (weighted arithmetic mean of normalized components) now applies uniformly to all six variables, eliminating the ad hoc mix of multiplication, subtraction, and division.

**Specific Revision Made:** Formula rewritten as a weighted arithmetic mean; the ad hoc original operators no longer appear in the working model (they are shown only for historical/audit purposes).

**Location in Revised Manuscript:** Methods, “Reconstructed formula (GEAF′)”.

***(b) Dimensional inconsistency between A×E and C×I.***

**Response:** Resolved: every variable is transformed to a dimensionless [0,1] component before combination, so no term compares or combines incompatible units.

**Specific Revision Made:** Added normalized components A_n, E_n, C_n, I_n, P_n, R_n with explicit transformation formulas.

**Location in Revised Manuscript:** Methods, “Reconstructed formula (GEAF′)”; Table 2 (parameter definitions).

***(c) Normalization of C is unspecified.***

**Response:** Resolved: C_n = 1 − min(C/C_max, 1), with C_max set as an explicit modeling normalization ceiling rather than an affordability claim.

**Specific Revision Made:** C_max = $4.25 million, anchored to the verified US wholesale acquisition cost of Lenmeldy (Orchard Therapeutics, FDA approval March 2024); sensitivity to this choice is stated as a limitation.

**Location in Revised Manuscript:** Methods, “Reconstructed formula (GEAF′)”; Table 2; Table 8 (Limitations).

***(d) The meaningful range/interpretation thresholds are not established.***

**Response:** Resolved: GEAF′ is bounded on [0,100] by construction, verified numerically at algebraic extremes; no unjustified clinical/policy “acceptability” threshold is asserted.

**Specific Revision Made:** Edge-case verification (worst case = 0.0, best case = 100.0) reported; color bands in Fig 2 explicitly labeled as visual reference only, not thresholds.

**Location in Revised Manuscript:** Results, “Edge-case behavior” subsection; Fig 2 caption.

***The manuscript is presented as a Research Article without meeting empirical methodological standards; reclassify as conceptual/methods paper or strengthen with empirical calibration and sensitivity analysis.***

**Response:** Both actions taken to the extent honestly possible: the manuscript is now explicitly framed as a conceptual/methodological framework paper throughout, and genuine one-way, two-way, and 10,000-iteration Monte Carlo sensitivity analysis was added. No empirical calibration against real-world outcome data is claimed, because none was performed.

**Specific Revision Made:** Framing language added to Abstract, Introduction (Research Questions), and Methods; full sensitivity/uncertainty analysis section added (previously only a single ±20% cost check existed).

**Location in Revised Manuscript:** Abstract; Introduction §1.1; Methods §3.5; Results §4.3-4.5; Table 8 lists empirical calibration as future work, not completed work.

***Ethical discussion is essayistic and superficial; philosophical frameworks under-analyzed; EEC/TMF/ISCA too abstract; ISSCR provisions not analyzed; many claims uncited.***

**Response:** The ethics/background section was shortened and refocused on material that motivates the quantitative model and the governance table; EEC/TMF/ISCA are now operationalized with explicit purpose, membership, decision process, and open questions, and labeled as non-binding proposals with no existing institutional standing.

**Specific Revision Made:** Conceptual Background condensed by roughly half; new governance table with purpose/scope/decision-process/institutional-status/limitations columns for each of EEC, TMF, and ISCA.

**Location in Revised Manuscript:** Conceptual Background section; Table 1.

***Scenario parameter values (A=20, E=0.6, I=0.7, etc.) have no documented empirical basis.***

**Response:** Every parameter's evidentiary status is now labeled explicitly as either a published estimate or an illustrative assumption, and every scenario cell carries a specific justification.

**Specific Revision Made:** New parameter-evidence table and per-scenario justification table.

**Location in Revised Manuscript:** Table 2 (parameter definitions and evidentiary status); Table 3 (scenario inputs and justification).

***Unsupported claim that targeted policies could reduce therapy costs “by up to 70%”; ±20%-on-cost-only sensitivity analysis is insufficient to support it.***

**Response:** The 70% figure, an associated 2035 global-average projection, and other unsupported forward-looking claims were removed, not softened. Cost reductions embedded in specific scenarios are explicitly labeled illustrative modeling assumptions, not empirical projections.

**Specific Revision Made:** Deletion of the 70% claim and related projections from Abstract, Results, Discussion, and Conclusion; explicit statement that a real cost-reduction claim would require therapy- and market-specific economic modeling not undertaken here.

**Location in Revised Manuscript:** Abstract; Results §4.2; Table 8 (Limitations).

***The affiliation pattern (eight institutions including professional societies and the author's own company/foundation) requires clarification.***

**Response:** Addressed directly: the affiliation is reduced to the single institution that provided the research environment for this work; the remaining seven entries did not meet PLOS ONE's affiliation standard.

**Specific Revision Made:** Affiliation line changed to Strayer University only; professional-society memberships moved to Acknowledgments as memberships; Eloi Holding, Inc. and David Oloche Foundation moved to Funding/Competing Interests/Acknowledgments as appropriate, not affiliations.

**Location in Revised Manuscript:** Title page; Acknowledgments; Funding Statement; Competing Interests.

***No structured comparison with the WHO framework, ECEA, fair-innings approach, or equity-weighted QALY/DALY models.***

**Response:** Added a structured comparison table against ICER, QALY/DALY-based HTA, DCEA, ECEA, and WHO health-equity assessment frameworks. A formal “fair-innings” row was deliberately omitted, as that framework addresses age-based prioritization rather than cost/access/ethics integration and is not a close conceptual comparator.

**Specific Revision Made:** New seven-row framework comparison table.

**Location in Revised Manuscript:** Table 7 (framework comparison).

***Minor: the title is hyperbolic (“Revolutionizing the Genome”).***

**Response:** Agreed; changed to a neutral, descriptive title.

**Specific Revision Made:** Title changed from “Revolutionizing the Genome: Ethical Innovations, Equitable Policies, and a Mathematical Framework for Affordable Regenerative Medicine” to “The Genomic Equity Accessibility Framework (GEAF): A Dimensionally Consistent Framework for Modeling Affordability and Equity in Genomic and Regenerative Therapies.”

**Location in Revised Manuscript:** Title page; running header of all three final documents.

***Minor: grammatical errors and imprecise/promotional language (e.g., “fostering a kinder and more inclusive future”).***

**Response:** Removed throughout; the manuscript now uses standard scientific register.

**Specific Revision Made:** Abstract, Discussion, and Conclusion rewritten without promotional phrasing; full-text audit confirmed no remaining instances.

**Location in Revised Manuscript:** Whole manuscript, particularly Abstract and Conclusion.

***Minor: an ‘LLM NOTE’ states no LLM was used in preparation, which may no longer apply.***

**Response:** Removed and replaced with an accurate AI/LLM Use Disclosure describing actual AI involvement and the author's verification and responsibility.

**Specific Revision Made:** New AI/LLM Use Disclosure paragraph.

**Location in Revised Manuscript:** Title page; Declarations.

***Minor: numerous typographical/formatting errors; inconsistent reference formatting (numbered vs. author-date).***

**Response:** The reference list was deduplicated (the original contained several references repeated 3-4 times under different numbers) and reformatted consistently.

**Specific Revision Made:** Reference list rebuilt as a single deduplicated, consistently formatted numbered list (32 entries, down from 95 with duplicates).

**Location in Revised Manuscript:** Final References section.

***Minor: Methods is embedded within Discussion rather than presented as a standalone section.***

**Response:** Resolved: Methods is now a standalone section preceding Results, in standard PLOS ONE order.

**Specific Revision Made:** Full structural reorganization into Introduction / Conceptual Background / Methods / Results / Discussion / Conclusion / Declarations / References.

**Location in Revised Manuscript:** Overall manuscript structure (Table of Contents-level).

***Minor: the seven scenarios lack a systematic rationale for parameter choices.***

**Response:** Resolved: every scenario cell now carries an explicit justification/evidence label.

**Specific Revision Made:** New scenario-justification table.

**Location in Revised Manuscript:** Table 3.

***Minor: add a limitations section acknowledging the conceptual nature, lack of empirical validation, and need for prospective testing.***

**Response:** Added a dedicated limitations section and table covering eight specific limitations and proposed mitigations.

**Specific Revision Made:** New Limitations subsection and table, including data-calibration needs, weighting-scheme assumptions, cost-ceiling sensitivity, and the compensatory-aggregation property.

**Location in Revised Manuscript:** Discussion §5.5; Table 8.

# Response to Reviewer #2

***Research questions/hypotheses are unclear; topics too broad; delete general ethics content not directly tied to GEAF.***

**Response:** Four explicit research questions and the study's scope/unit of analysis are now stated; the ethics/background section is shortened and reorganized so every retained paragraph connects to the quantitative model or governance table.

**Specific Revision Made:** New Research Questions and Scope subsections added; Conceptual Background condensed by roughly half.

**Location in Revised Manuscript:** Introduction §1.1-1.2; Conceptual Background section.

***Figures are conceptual only; original quantitative charts are needed (multi-scenario comparison, tornado, sensitivity, GEAF-vs-ICER).***

**Response:** Five original, model-derived quantitative figures were added. A GEAF-vs-ICER chart and a population-impact projection chart were not added, because no shared dataset or non-fabricated projection basis exists for either.

**Specific Revision Made:** Fig 2 (scenario comparison bar chart), Fig 3 (tornado sensitivity), Fig 4 and Fig 5 (two-way sensitivity heatmaps), Fig 6 (Monte Carlo distributions), all generated from the manuscript's own model via the code in S1 Appendix.

**Location in Revised Manuscript:** Results §4.2-4.5 and their figure captions; absence of a GEAF-vs-ICER chart explicitly flagged in §4.6.

***The ethical statement is too general; it should state that no individual-level data were used and discuss governance principles for genomic data sharing.***

**Response:** Explicit statement added that no human participants, animal subjects, identifiable genomic data, or biological material were used, plus a separate discussion of governance principles (privacy, consent, community governance, benefit-sharing) relevant to any future implementation of proposals such as the Global Genomic Commons.

**Specific Revision Made:** New Ethics Approval and Data Governance subsection.

**Location in Revised Manuscript:** Methods §3.6.

***Abbreviations are not defined at first use (DCEA, HTA, SCBEM, etc.); colloquial language in formulas/policy sections; overly long paragraphs.***

**Response:** All abbreviations are now defined at first use; the formula and Methods sections use formal quantitative language; long paragraphs were split and reorganized.

**Specific Revision Made:** Full-text abbreviation audit; Methods rewritten in formal register; Conceptual Background paragraphs shortened and split.

**Location in Revised Manuscript:** Whole manuscript, particularly Methods and Conceptual Background.

***Need disease/region-specific epidemiological data (e.g., sickle cell disease diagnosis gap in Africa; Asia gene-therapy coverage); cite 2024-2025 WHO African genomic report and Nigerian sickle-cell survey data.***

**Response:** The WHO (2025) finding on the concentration of clinical genomic research in high-income countries and general LMIC infrastructure constraints is retained and used to justify one scenario's parameters. A Nigeria-specific quantitative table was not added, because no specific dataset matching the request could be independently verified within this revision; fabricating one would be a scientific-integrity violation.

**Specific Revision Made:** WHO (2025) finding retained and cited as justification for Scenario S4's parameter choices; no fabricated Nigeria-specific table added.

**Location in Revised Manuscript:** Conceptual Background; Table 3, Scenario S4 justification column.

***Add Wang et al., Gut Microbes 2026 (gut microbiota metabolic reprogramming).***

**Response:** Evaluated and not added: this reference concerns host metabolic-disease mechanisms via gut microbiota, with no substantive connection to genomic-therapy pricing, accessibility, or the GEAF′ index.

**Specific Revision Made:** Not added to the reference list.

**Location in Revised Manuscript:** Not applicable (reference deliberately excluded).

***Add Wu et al., Interdisciplinary Medicine 2026 (SUMOylation in tumorigenesis).***

**Response:** Evaluated and not added, for the same reason: a molecular oncology mechanism review unrelated to equity/affordability modeling.

**Specific Revision Made:** Not added to the reference list.

**Location in Revised Manuscript:** Not applicable (reference deliberately excluded).

***Add a functional foods/MAFLD gut-liver axis review.***

**Response:** Evaluated and not added, for the same reason: unrelated to genomic/regenerative therapy pricing or equity modeling.

**Specific Revision Made:** Not added to the reference list.

**Location in Revised Manuscript:** Not applicable (reference deliberately excluded).

***Peer-review identity/publication-preference question.***

**Response:** This is a submission-system administrative question for the corresponding author to answer directly in Editorial Manager; it does not require a manuscript change.

**Specific Revision Made:** No manuscript action required.

**Location in Revised Manuscript:** Not applicable.

# Post-Review Editorial and Disclosure Corrections (author-initiated, not reviewer-requested)

Three further corrections were made that were not requested by either reviewer but were necessary for scientific accuracy and PLOS ONE compliance. They are documented here for full transparency and are reflected throughout the final manuscript and the Original-to-Final Track Changes document.

***Reviewer-conversation language throughout the scientific manuscript (“Reviewer #1,” “Reviewer #2,” “the Academic Editor,” “this revision,” “for resubmission”).***

**Response:** Removed from the scientific manuscript and, where the underlying point was scientifically legitimate, rewritten as neutral scholarly prose; such language is retained only in this Response to Reviewers document.

**Specific Revision Made:** Full-text audit and rewrite of Introduction, Conceptual Background, Methods, Results, Discussion, and Conclusion.

**Location in Revised Manuscript:** Whole manuscript; verified by full-text search showing zero remaining reviewer-process references outside this document.

***Funding Statement incorrectly stated “no specific external funding” and Competing Interests incorrectly stated the funder had no involvement.***

**Response:** Corrected upon author confirmation that Eloi Holding, Inc. provided partial financial support. The standard “funder had no role” sentence is not used, since the author is both the funder's Founder and the sole researcher and these roles are not separable; this is stated explicitly rather than using an inaccurate boilerplate.

**Specific Revision Made:** Funding Statement and Competing Interests sections rewritten to disclose the funding relationship and the author's Founder role accurately.

**Location in Revised Manuscript:** Declarations – Funding; Declarations – Competing Interests.

***Monte Carlo 90% interval was reported using the 10th-90th percentile range (an 80% interval) rather than the 5th-95th percentile range (the correct 90% interval); Cmax was misattributed to Casgevy; the S1 baseline cost was justified as an incorrect arithmetic mean.***

**Response:** All three corrected upon internal verification: Monte Carlo intervals now correctly use the 5th-95th percentile; Cmax is correctly attributed to Lenmeldy (Orchard Therapeutics, 2024); the S1 baseline cost is now anchored to the verified Hemgenix (CSL Behring, 2022) list price rather than an incorrect calculated average. Means and medians were already correct throughout.

**Specific Revision Made:** Numerical corrections applied consistently in Abstract, Methods, Results, all affected tables, and S1 Appendix code comments.

**Location in Revised Manuscript:** Abstract; Methods §3.2, §3.4, §3.5; Results §4.5; Table 2; Table 3; Table 4; Table 8.

# Summary of Reviewer Requests Not Fulfilled As Literally Worded, and Why

In keeping with the principle that scientific integrity takes priority over satisfying a request literally, three items were not fulfilled as worded:

(i) A direct empirical GEAF′-versus-ICER comparison on real data was not performed, because no shared dataset reporting both sets of parameters for the same therapy-jurisdiction pair was identified (Results §4.6).

(ii) Nigeria/Africa-specific disease-level statistical tables were not added, because no specific dataset matching the request could be independently verified (Response to Reviewer #2, Item 5).

(iii) Three reviewer-suggested references (gut microbiota metabolic reprogramming; SUMOylation in oncology; functional foods/MAFLD) were evaluated and not included, as none are substantively relevant to a genomic-therapy equity-index methods paper (Response to Reviewer #2, Items 6-8).

All other reviewer and editor points have been addressed with specific, traceable manuscript changes as detailed above, each cross-checked against the final manuscript and the accompanying Original-to-Final Track Changes document.
