# Supplementary material for: The Genomic Equity Accessibility Framework (GEAF): A dimensionally consistent framework for modeling affordability and equity in genomic and regenerative therapies
Source: PLoS One. 2026 Oct 6;21(10):e0359782. doi: 10.1371/journal.pone.0359782 (PMC13641711; doi:10.1371/journal.pone.0359782)
Supplement: Peer review history

# Peer review: Decision Letter 0

**DOI:** 10.1371/journal.pone.0359782.r001

**Authors:** Kangkang Ji

25 Aug 2026

Dear Dr. David,

Thank you for submitting your manuscript to PLOS One. After careful consideration, we feel that it has merit but does not fully meet PLOS One’s publication criteria as it currently stands. Therefore, we invite you to submit a revised version of the manuscript that addresses the points raised during the review process.

Please submit your revised manuscript by Oct 24 2026 11:59PM. If you will need more time than this to complete your revisions, please reply to this message or contact the journal office at plosone@plos.org. When you're ready to submit your revision, log on to https://www.editorialmanager.com/pone/ and select the 'Submissions Needing Revision' folder to locate your manuscript file.

- A letter that responds to each point raised by the academic editor and reviewer(s). You should upload this letter as a separate file labeled 'Response to Reviewers'.
- A marked-up copy of your manuscript that highlights changes made to the original version. You should upload this as a separate file labeled 'Revised Manuscript with Track Changes'.
- An unmarked version of your revised paper without tracked changes. You should upload this as a separate file labeled 'Manuscript'.

If you would like to make changes to your financial disclosure, please include your updated statement in your cover letter. Guidelines for resubmitting your figure files are available below the reviewer comments at the end of this letter.

If applicable, we recommend that you deposit your laboratory protocols in protocols.io to enhance the reproducibility of your results. Protocols.io assigns your protocol its own identifier (DOI) so that it can be cited independently in the future. For instructions see: https://journals.plos.org/plosone/s/submission-guidelines#loc-laboratory-protocols. Additionally, PLOS One offers an option for publishing peer-reviewed Lab Protocol articles, which describe protocols hosted on protocols.io. Read more information on sharing protocols at https://plos.org/protocols?utm_medium=editorial-email&utm_source=authorletters&utm_campaign=protocols.

As the corresponding author, your ORCID iD is verified in the submission system and will appear in the published article. PLOS supports the use of ORCID, and we encourage all coauthors to register for an ORCID iD and use it as well. Please encourage your coauthors to verify their ORCID iD within the submission system before final acceptance, as unverified ORCID iDs will not appear in the published article. *Only*  the individual author can complete the verification step; PLOS staff *cannot*  verify ORCID iDs on behalf of authors.

We look forward to receiving your revised manuscript.

Kind regards,

Kangkang Ji, Ph.D., M.D.

Academic Editor

PLOS One

Journal Requirements:

When submitting your revision, we need you to address these additional requirements.

1. Please ensure that your manuscript meets PLOS ONE's style requirements, including those for file naming. The PLOS ONE style templates can be found at

https://journals.plos.org/plosone/s/file?id=wjVg/PLOSOne_formatting_sample_main_body.pdf and https://journals.plos.org/plosone/s/file?id=ba62/PLOSOne_formatting_sample_title_authors_affiliations.pdf

2. Please provide a complete Data Availability Statement in the submission form, ensuring you include all necessary access information or a reason for why you are unable to make your data freely accessible. If your research concerns only data provided within your submission, please write "All data are in the manuscript and/or supporting information files" as your Data Availability Statement.

3. Please update your submission to use the PLOS LaTeX template. The template and more information on our requirements for LaTeX submissions can be found at http://journals.plos.org/plosone/s/latex.

4. Please amend your list of authors on the manuscript to ensure that each author is linked to an affiliation. Authors’ affiliations should reflect the institution where the work was done (if authors moved subsequently, you can also list the new affiliation stating “current affiliation:….” as necessary).

5. If the reviewer comments include a recommendation to cite specific previously published works, please review and evaluate these publications to determine whether they are relevant and should be cited. There is no requirement to cite these works unless the editor has indicated otherwise.

[Note: HTML markup is below. Please do not edit.]

Reviewers' comments:

Reviewer's Responses to Questions

**Comments to the Author**

1. Is the manuscript technically sound, and do the data support the conclusions?

Reviewer #1: Yes

Reviewer #2: Yes

**********

2. Has the statistical analysis been performed appropriately and rigorously?

Reviewer #1: Yes

Reviewer #2: Yes

**********

3. Have the authors made all data underlying the findings in their manuscript fully available?

The PLOS Data policy

Reviewer #1: Yes

Reviewer #2: Yes

**********

4. Is the manuscript presented in an intelligible fashion and written in standard English?

Reviewer #1: Yes

Reviewer #2: Yes

**********

Reviewer #1: This manuscript presents a conceptual essay proposing the Genomic Equity Accessibility Formula (GEAF), a mathematical framework for evaluating and improving the affordability and equitable distribution of genomic and regenerative therapies. The paper addresses important topics — CRISPR gene editing ethics, synthetic embryo models, stem cell research regulation, and health equity in genomic medicine — and proposes policy frameworks including Ethical Enhancement Councils (EECs), a Tiered Moral Framework (TMF), and International Stem Cell Accords (ISCAs). The ambition to bridge bioethics with quantitative modeling is commendable. However, the manuscript suffers from significant methodological and presentational shortcomings that substantially limit its scholarly contribution. The GEAF formula, while mathematically simple, lacks rigorous derivation, validation against empirical data, and sensitivity analysis. The manuscript reads more as a policy opinion piece than a research article, and the connection between the qualitative ethical discussion (which constitutes the majority of the text) and the quantitative GEAF model is insufficiently developed.

The Introduction broadly surveys CRISPR, synthetic embryos, stem cell regulation, and health equity challenges. The authors should consider acknowledging the intersection between genomics policy and the food-medicine homology paradigm, where natural products are increasingly studied using genomic and multi-omics approaches. The review by Zhang et al. (DOI: 10.26599/FMH.2025.9420059) on Panax ginseng for medicinal and edible value demonstrates how genomic tools are being applied to characterize complex botanical therapeutics — an application domain that raises parallel questions about equitable access to traditionally-used medicinal resources. The review by Li et al. (DOI: 10.26599/FMH.2025.9420120) on food and medicine homology in disease treatment provides a conceptual framework relevant to how regulatory frameworks and equitable access policies in one domain (traditional medicine/food-medicine homology) may inform approaches in another (personalized genomic medicine).

1. The most significant concern is the GEAF formula itself, which constitutes the claimed methodological contribution. The formula GEAF = ((A × E) − (C × I)) / (P + R) × 100 lacks rigorous theoretical justification. Several fundamental issues must be addressed: (a) The linear additive/multiplicative structure is arbitrary — why multiply A×E rather than using a weighted sum? Why subtract C×I rather than dividing by it? The authors must provide a principled derivation, potentially grounded in multi-attribute utility theory, health economics frameworks, or established equity metrics; (b) Dimensional analysis reveals internal inconsistencies — A (percentage, 0-100) multiplied by E (0-1) yields units incompatible with C (cost in thousands of USD) multiplied by I (Gini-like coefficient, 0-1). The subtraction of incommensurate quantities is mathematically improper; (c) The normalization approach is unclear — C is described as "normalized USD value" but the method of normalization is not specified; (d) The formula produces negative values (e.g., GEAF = -105.63) which are interpreted as "inequities," but the meaningful range and interpretation thresholds are not established — at what GEAF value is equity considered "acceptable"?

2. The study is presented as a "Research Article" with defined Methods, Results, and Discussion sections. However, it does not meet the methodological standards of empirical research. The "Methods" describe a "conceptual and mathematical modeling approach" using "manual calculations and scenario-based simulations without specialized software." No empirical data were collected, no statistical analyses were performed, and the seven "scenarios" consist of manually chosen input values with no systematic approach to parameter space exploration. The authors should either: (a) substantially strengthen the GEAF framework with formal derivation, empirical calibration against real-world health equity datasets (e.g., global gene therapy access statistics, WHO health equity indicators, country-level pharmaceutical expenditure data), systematic sensitivity analysis (e.g., Monte Carlo simulation), and validation against known equity outcomes; or (b) reclassify the manuscript as a Perspective/Opinion piece and reframe the GEAF as a conceptual heuristic rather than a validated quantitative model.

3. The ethical discussion sections (covering CRISPR, synthetic embryos, stem cell regulation) are written in an essayistic style without systematic literature review methodology. Major concerns include: (a) Key ethical frameworks are mentioned superficially — deontology, consequentialism, and procreative beneficence are each discussed in only 1-2 sentences without substantive analysis of how these frameworks reach divergent conclusions on specific applications; (b) The proposed policy innovations (EECs, TMF, ISCAs) are described at a high level of abstraction without operational detail. For example, the Tiered Moral Framework proposes that "pre-implantation models can be used freely" while "post-gastrulation models need justification" — but what constitutes "justification"? Who adjudicates? What criteria apply?; (c) The ISSCR 2025 guidelines are referenced multiple times but the specific provisions relevant to the authors' proposals are not analyzed in detail; (d) Many claims lack supporting citations.

4. The GEAF scenario analysis (Section Results) relies entirely on "hypothetical data based on real-world estimates," yet the sources and derivation of these parameter values are not documented. For example, in the "Baseline Scenario": A=20 — what is the empirical basis for this value? Is it derived from WHO data on gene therapy coverage in LMICs?; E=0.6 — how is this quantified from real-world regulatory data?; I=0.7 — how is this "Gini-like coefficient" computed from actual health access disparities? Without transparent parameter derivation, the scenarios are essentially numerical illustrations of the formula's algebraic properties rather than meaningful policy analyses. Each parameter must be anchored to citable empirical data or a documented estimation protocol.

5. The manuscript claims that "targeted actions...could lower therapy costs by up to 70% for low-income groups" (Abstract). This claim appears in the Abstract and Discussion as a key finding, but the Methods do not describe how this 70% figure was derived. The sensitivity analysis consists only of adjusting cost (C) by ±20% in the baseline scenario, which is insufficient to support the 70% cost reduction claim. Either: (a) provide a detailed economic model demonstrating the cost reduction mechanisms with quantitative estimates grounded in health economics literature; or (b) remove unsupported quantitative claims and present cost reduction as a conceptual aspiration.

6. The manuscript is presented as having a single author with affiliations listed across eight institutions spanning the US, UK, and Nigeria. This unusual affiliation pattern requires clarification: (a) Which institution(s) provided the primary intellectual environment for this work? (b) What is the nature of the author's relationship with each listed entity? (c) Several affiliations appear unrelated to the manuscript's content. The journal's authorship and affiliation policies should be reviewed to ensure compliance.

7. The manuscript would benefit from a structured comparison with existing health equity frameworks and quantitative models. The authors mention cost-effectiveness models (ICERs) and multi-attribute utility theory in passing but do not systematically compare GEAF against: (a) the WHO health equity assessment framework; (b) extended cost-effectiveness analysis (ECEA) methods used in global health; (c) the fair innings approach and other distributive justice frameworks for healthcare resource allocation; and (d) existing equity-weighted QALY/DALY models.

Minor points:

The title "Revolutionizing the Genome" is hyperbolic for a conceptual modeling paper and does not accurately describe the content.

The Abstract contains grammatical errors and imprecise language (e.g., "fostering a kinder and more inclusive future"). Scientific manuscripts should maintain professional, objective language.

A "LLM NOTE" stating "No large language model use in preparation of this research paper" appears in the manuscript front matter. If required by the journal, the format should comply with journal policy. If not required, it should be removed.

The manuscript contains numerous typographical, formatting, and grammatical errors throughout that require thorough proofreading.

The reference formatting is inconsistent. Some citations use numbered references while others use author-date format.

The manuscript organization is unconventional for a research article: the Methods section is embedded within the Discussion narrative rather than appearing as a standalone section before Results.

The seven GEAF scenarios in Table 1 are presented without a systematic rationale for the parameter choices.

The manuscript states "No datasets were generated or analysed during the current study" which is appropriate for a conceptual paper but should be clearly reflected in the Methods section.

Consider adding a limitations section that frankly acknowledges: the conceptual nature of GEAF, the lack of empirical validation, and the need for prospective testing against real-world policy outcomes.

Reviewer #2: Revolutionizing the Genome: Ethical Innovations, Equitable Policies, and a

Mathematical Framework for Affordable Regenerative Medicine

1. The research questions and main hypotheses are not clear enough. Currently, various topics such as non-treatment gene editing, synthetic embryo models, stem cell regulation, global health equity, and payment policies are being discussed simultaneously, which is too broad and lacks a testable central issue. It is suggested to clearly define the main research questions, secondary questions, research subjects, analysis units, and testable hypotheses in the introduction section, and to delete the general ethical descriptions that are not directly related to GEAF.

2. The 7 figures in the text only provide basic conceptual displays, without original quantitative analysis charts; there is a lack of multi-scenario comparison line graphs of GEAF, tornado charts of sensitivity analysis, and bar charts of fair benefits from different policy interventions. The visual support is seriously insufficient. It is suggested to add 4 types of original quantitative charts: ① Comparison charts of GEAF scores for 7 scenarios; ② Dual-factor tornado charts of sensitivity analysis of cost C and inequality factor I; ③ Prediction charts of global accessible population changes before and after EGI intervention; ④ Comparison charts of GEAF and traditional ICER evaluation results, which should be placed in the main text or supplementary materials.

3. The 7 figures in the text only provide basic conceptual displays, without original quantitative analysis charts; there is a lack of multi-scenario comparison line graphs of GEAF, tornado charts of sensitivity analysis, and bar charts of fair benefits from different policy interventions. The visual support is seriously insufficient. It is suggested to add 4 types of original quantitative charts: ① Comparison charts of GEAF scores for 7 scenarios; ② Dual-factor tornado charts of sensitivity analysis of cost C and inequality factor I; ③ Prediction charts of global accessible population changes before and after EGI intervention; ④ Comparison charts of GEAF and traditional ICER evaluation results, which should be placed in the main text or supplementary materials.

4. The ethical statement is too general. Although the study does not involve human or animal subjects, it involves issues of human genome equity, vulnerable groups, and data governance. It is not sufficient to simply use "N/A" to replace the ethical position explanation. It is suggested to clearly state in the methods or discussion section that this study does not contain individual-level data, and to explain the ethical principles and potential risks followed when involving genomic data sharing and policy recommendations.

5. The first appearance of some professional term abbreviations in the full text is not marked with the full name, such as DCEA, HTA, SCBEM; the long sentences are excessively piled up, and the paragraph logic jumps abruptly; some policies and mathematical formulas are expressed in colloquial language, which does not conform to the academic writing norms of PLOS One. It is suggested to uniformly check all abbreviations in the full text, mark the full English names for the first appearance; split extremely long paragraphs, and rationalize the internal argument logic of the paragraphs; revise the formulas and policy explanations to use more formal language, eliminate redundant background scientific explanations, and focus on the original research analysis.

6. The inequality of medical accessibility only lists the bar charts of prices for high-income/LMIC regions, lacking real epidemiological data support for specific diseases and regions, such as the gap in diagnosis and treatment of sickle cell anemia in Africa, and the coverage rate of rare disease gene therapies in Asia without specific population statistics. It is suggested to supplement large continent and disease-specific statistical tables of genomic diagnosis accessibility; cite the 2024–2025 WHO African genomic report and the real data from the Nigerian sickle cell anemia survey to quantify the scale of unmet medical needs.

7. It is suggested to add references: Wang, Y., Huang, B., Wei, X., Guan, Y., Li, L.*, Zheng, Y., Sun, W. Gut Microbiota Metabolic Reprogramming Drives the Development of Metabolic Diseases in the Host. Gut Microbes, 2026, 18 (1), 2644681. This document is an authoritative review in the field of intestinal microbiota in 2026, systematically explaining the complete molecular pathway by which the metabolic reprogramming of the intestinal microbiota induces host metabolic diseases. It is highly consistent with the research topic of this article, providing a cutting-edge literature support for the discussion of mechanisms, and completing the core research progress in the field this year, enhancing the completeness of the discussion.

8. Suggested supplementary citation: Yimao Wu, Zichang Chen, Yinting Hu, Gokhan Zengin, Qian Zhang*, Meng-Yao Li*, Wenlong Sun*. SUMOylation Regulates Tumorigenesis and Progression: Molecular Mechanisms and Therapeutic Applications, Interdisciplinary Medicine, 2026;e70120. This document is a latest interdisciplinary review in 2026, systematically summarizing the complete molecular pathway of SUMOylation modification regulating tumor occurrence and progression and its therapeutic potential. It is highly compatible with the research topic of this article, providing a cutting-edge literature support for the discussion of mechanisms, completing the core research progress in the field this year, and enhancing the timeliness and completeness of the discussion.

9. Suggested supplementary citation: Potential effects of functional foods and dietary supplements on metabolic-associated fatty liver disease and the underlying mechanisms: a narrative review with a focus on the modulation of the gut microbiota. This review focuses on the complete action pathway of functional foods and dietary supplements in modulating metabolic-associated fatty liver disease (MAFLD) and systematically summarizes the lipid metabolism and inflammatory regulation mechanisms mediated by the gut-liver axis, which is highly compatible with the core direction of this research. It can supplement the latest consensus in the field, improve the literature support for the mediation of dietary intervention through the intestinal microbiota in improving fatty liver, and enhance the completeness and timeliness of the discussion.

**********

what does this mean?). If published, this will include your full peer review and any attached files.

If you choose “no”, your identity will remain anonymous but your review may still be made public.

**Do you want your identity to be public for this peer review?**  For information about this choice, including consent withdrawal, please see our Privacy Policy

Reviewer #1: No

Reviewer #2: No

**********

[NOTE: If reviewer comments were submitted as an attachment file, they will be attached to this email and accessible via the submission site. Please log into your account, locate the manuscript record, and check for the action link "View Attachments". If this link does not appear, there are no attachment files.]

To ensure your figures meet our technical requirements, please review our figure guidelines: https://journals.plos.org/plosone/s/figures

You may also use PLOS’s free figure tool, NAAS, to help you prepare publication quality figures: https://journals.plos.org/plosone/s/figures#loc-tools-for-figure-preparation.

NAAS will assess whether your figures meet our technical requirements by comparing each figure against our figure specifications.

# Author comment: Author response to Decision Letter 1

**DOI:** 10.1371/journal.pone.0359782.r002

12 Sep 2026

Final Response to Reviewers

Manuscript: PONE-D-26-32218. Original title: “Revolutionizing the Genome: Ethical Innovations, Equitable Policies, and a Mathematical Framework for Affordable Regenerative Medicine.” Final title: “The Genomic Equity Accessibility Framework (GEAF): A Dimensionally Consistent Framework for Modeling Affordability and Equity in Genomic and Regenerative Therapies.”

We thank the Academic Editor and both reviewers for a thorough, constructive review. The central and correct criticism - that the original GEAF formula was mathematically unsound and insufficiently justified - has been addressed by reconstructing the index from first principles (Methods, Model Reconstruction) rather than defending the original formulation. Each point below follows a consistent structure: the reviewer's comment, our response, the specific revision made, and its exact location in the final manuscript. We are explicit about the small number of requests we did not fulfill as literally worded (a direct empirical GEAF-vs-ICER comparison; Nigeria-specific epidemiological tables; three reviewer-suggested off-topic citations), and state why in each case.

Response to the Academic Editor

Please ensure the manuscript meets PLOS ONE style requirements, including file naming.

Response: The manuscript follows PLOS ONE's structural conventions (title page, structured Abstract, standalone Methods before Results, Declarations) and this response follows PLOS ONE's file-naming convention.

Specific Revision Made: Full structural reorganization; consistent PLOS ONE file naming applied to all three final documents.

Location in Revised Manuscript: Whole-manuscript structure; filenames GEAF_PONE_D_26_32218_Final_Clean_Manuscript.docx, GEAF_PONE_D_26_32218_Original_to_Final_Track_Changes_Reviewers.docx, GEAF_PONE_D_26_32218_Final_Response_to_Reviewers.docx.

Please provide a complete Data Availability Statement.

Response: Added a complete Data Availability Statement appropriate to a conceptual/modeling study.

Specific Revision Made: “All data are in the manuscript and/or supporting information files”, with all parameters, scenarios, and analysis code disclosed in-text and in S1 Appendix.

Location in Revised Manuscript: Declarations – Data Availability Statement.

Please update the submission to the PLOS LaTeX template.

Response: Noted for final typesetting; the DOCX content is structured so that a PLOS LaTeX conversion at final submission is straightforward.

Specific Revision Made: No manuscript content change required; this is a production-format step to be completed at upload.

Location in Revised Manuscript: Not applicable to manuscript text.

Please amend the author list so each author is linked to an affiliation.

Response: The manuscript has a single author; affiliation is corrected to reflect only the institution that provided the research environment for this work.

Specific Revision Made: Affiliation reduced from eight listed institutional addresses to Strayer University alone; professional-society memberships and the author's own organizations (Eloi Holding, Inc.; David Oloche Foundation) are disclosed separately as memberships/funding/competing interests, not affiliations.

Location in Revised Manuscript: Title page (affiliation line); Acknowledgments; Funding Statement; Competing Interests.

If reviewers recommend citing specific works, evaluate relevance; no obligation to include.

Response: Each reviewer-suggested reference was evaluated individually and included only where substantively relevant.

Specific Revision Made: Three reviewer-suggested references (gut microbiota metabolic reprogramming; SUMOylation in oncology; functional foods/MAFLD) were evaluated and not included, as none bear on a genomic-therapy equity-index methods paper.

Location in Revised Manuscript: See Reviewer #2, Items 7-9 below; final Reference list.

Response to Reviewer #1

The GEAF formula lacks rigorous theoretical justification; the multiplicative/subtractive structure is arbitrary.

Response: Agreed in full. The original formula was audited against four validity criteria (dimensional consistency, boundedness, denominator behavior, scenario-formula consistency) and found deficient on all four; it was reconstructed rather than defended.

Specific Revision Made: New Methods subsection auditing the original formula's defects, followed by a full reconstruction using multi-attribute utility theory: GEAF' = 100 × Σ(wᵢ × Xᵢ), six components normalized to [0,1], equal weights (1/6) as a stated default.

Location in Revised Manuscript: Methods, “Audit of the originally proposed formula” and “Reconstructed formula (GEAF′)” subsections.

(a) Why multiply A×E rather than a weighted sum? Why subtract C×I rather than divide?

Response: Resolved by reconstruction: a single, consistent aggregation rule (weighted arithmetic mean of normalized components) now applies uniformly to all six variables, eliminating the ad hoc mix of multiplication, subtraction, and division.

Specific Revision Made: Formula rewritten as a weighted arithmetic mean; the ad hoc original operators no longer appear in the working model (they are shown only for historical/audit purposes).

Location in Revised Manuscript: Methods, “Reconstructed formula (GEAF′)”.

(b) Dimensional inconsistency between A×E and C×I.

Response: Resolved: every variable is transformed to a dimensionless [0,1] component before combination, so no term compares or combines incompatible units.

Specific Revision Made: Added normalized components A_n, E_n, C_n, I_n, P_n, R_n with explicit transformation formulas.

Location in Revised Manuscript: Methods, “Reconstructed formula (GEAF′)”; Table 2 (parameter definitions).

(c) Normalization of C is unspecified.

Response: Resolved: C_n = 1 − min(C/C_max, 1), with C_max set as an explicit modeling normalization ceiling rather than an affordability claim.

Specific Revision Made: C_max = $4.25 million, anchored to the verified US wholesale acquisition cost of Lenmeldy (Orchard Therapeutics, FDA approval March 2024); sensitivity to this choice is stated as a limitation.

Location in Revised Manuscript: Methods, “Reconstructed formula (GEAF′)”; Table 2; Table 8 (Limitations).

(d) The meaningful range/interpretation thresholds are not established.

Response: Resolved: GEAF′ is bounded on [0,100] by construction, verified numerically at algebraic extremes; no unjustified clinical/policy “acceptability” threshold is asserted.

Specific Revision Made: Edge-case verification (worst case = 0.0, best case = 100.0) reported; color bands in Fig 2 explicitly labeled as visual reference only, not thresholds.

Location in Revised Manuscript: Results, “Edge-case behavior” subsection; Fig 2 caption.

The manuscript is presented as a Research Article without meeting empirical methodological standards; reclassify as conceptual/methods paper or strengthen with empirical calibration and sensitivity analysis.

Response: Both actions taken to the extent honestly possible: the manuscript is now explicitly framed as a conceptual/methodological framework paper throughout, and genuine one-way, two-way, and 10,000-iteration Monte Carlo sensitivity analysis was added. No empirical calibration against real-world outcome data is claimed, because none was performed.

Specific Revision Made: Framing language added to Abstract, Introduction (Research Questions), and Methods; full sensitivity/uncertainty analysis section added (previously only a single ±20% cost check existed).

Location in Revised Manuscript: Abstract; Introduction §1.1; Methods §3.5; Results §4.3-4.5; Table 8 lists empirical calibration as future work, not completed work.

Ethical discussion is essayistic and superficial; philosophical frameworks under-analyzed; EEC/TMF/ISCA too abstract; ISSCR provisions not analyzed; many claims uncited.

Response: The ethics/background section was shortened and refocused on material that motivates the quantitative model and the governance table; EEC/TMF/ISCA are now operationalized with explicit purpose, membership, decision process, and open questions, and labeled as non-binding proposals with no existing institutional standing.

Specific Revision Made: Conceptual Background condensed by roughly half; new governance table with purpose/scope/decision-process/institutional-status/limitations columns for each of EEC, TMF, and ISCA.

Location in Revised Manuscript: Conceptual Background section; Table 1.

Scenario parameter values (A=20, E=0.6, I=0.7, etc.) have no documented empirical basis.

Response: Every parameter's evidentiary status is now labeled explicitly as either a published estimate or an illustrative assumption, and every scenario cell carries a specific justification.

Specific Revision Made: New parameter-evidence table and per-scenario justification table.

Location in Revised Manuscript: Table 2 (parameter definitions and evidentiary status); Table 3 (scenario inputs and justification).

Unsupported claim that targeted policies could reduce therapy costs “by up to 70%”; ±20%-on-cost-only sensitivity analysis is insufficient to support it.

Response: The 70% figure, an associated 2035 global-average projection, and other unsupported forward-looking claims were removed, not softened. Cost reductions embedded in specific scenarios are explicitly labeled illustrative modeling assumptions, not empirical projections.

Specific Revision Made: Deletion of the 70% claim and related projections from Abstract, Results, Discussion, and Conclusion; explicit statement that a real cost-reduction claim would require therapy- and market-specific economic modeling not undertaken here.

Location in Revised Manuscript: Abstract; Results §4.2; Table 8 (Limitations).

The affiliation pattern (eight institutions including professional societies and the author's own company/foundation) requires clarification.

Response: Addressed directly: the affiliation is reduced to the single institution that provided the research environment for this work; the remaining seven entries did not meet PLOS ONE's affiliation standard.

Specific Revision Made: Affiliation line changed to Strayer University only; professional-society memberships moved to Acknowledgments as memberships; Eloi Holding, Inc. and David Oloche Foundation moved to Funding/Competing Interests/Acknowledgments as appropriate, not affiliations.

Location in Revised Manuscript: Title page; Acknowledgments; Funding Statement; Competing Interests.

No structured comparison with the WHO framework, ECEA, fair-innings approach, or equity-weighted QALY/DALY models.

Response: Added a structured comparison table against ICER, QALY/DALY-based HTA, DCEA, ECEA, and WHO health-equity assessment frameworks. A formal “fair-innings” row was deliberately omitted, as that framework addresses age-based prioritization rather than cost/access/ethics integration and is not a close conceptual comparator.

Specific Revision Made: New seven-row framework comparison table.

Location in Revised Manuscript: Table 7 (framework comparison).

Minor: the title is hyperbolic (“Revolutionizing the Genome”).

Response: Agreed; changed to a neutral, descriptive title.

Specific Revision Made: Title changed from “Revolutionizing the Genome: Ethical Innovations, Equitable Policies, and a Mathematical Framework for Affordable Regenerative Medicine” to “The Genomic Equity Accessibility Framework (GEAF): A Dimensionally Consistent Framework for Modeling Affordability and Equity in Genomic and Regenerative Therapies.”

Location in Revised Manuscript: Title page; running header of all three final documents.

Minor: grammatical errors and imprecise/promotional language (e.g., “fostering a kinder and more inclusive future”).

Response: Removed throughout; the manuscript now uses standard scientific register.

Specific Revision Made: Abstract, Discussion, and Conclusion rewritten without promotional phrasing; full-text audit confirmed no remaining instances.

Location in Revised Manuscript: Whole manuscript, particularly Abstract and Conclusion.

Minor: an ‘LLM NOTE’ states no LLM was used in preparation, which may no longer apply.

Response: Removed and replaced with an accurate AI/LLM Use Disclosure describing actual AI involvement and the author's verification and responsibility.

Specific Revision Made: New AI/LLM Use Disclosure paragraph.

Location in Revised Manuscript: Title page; Declarations.

Minor: numerous typographical/formatting errors; inconsistent reference formatting (numbered vs. author-date).

Response: The reference list was deduplicated (the original contained several references repeated 3-4 times under different numbers) and reformatted consistently.

Specific Revision Made: Reference list rebuilt as a single deduplicated, consistently formatted numbered list (32 entries, down from 95 with duplicates).

Location in Revised Manuscript: Final References section.

Minor: Methods is embedded within Discussion rather than presented as a standalone section.

Response: Resolved: Methods is now a standalone section preceding Results, in standard PLOS ONE order.

Specific Revision Made: Full structural reorganization into Introduction / Conceptual Background / Methods / Results / Discussion / Conclusion / Declarations / References.

Location in Revised Manuscript: Overall manuscript structure (Table of Contents-level).

Minor: the seven scenarios lack a systematic rationale for parameter choices.

Response: Resolved: every scenario cell now carries an explicit justification/evidence label.

Specific Revision Made: New scenario-justification table.

Location in Revised Manuscript: Table 3.

Minor: add a limitations section acknowledging the conceptual nature, lack of empirical validation, and need for prospective testing.

Response: Added a dedicated limitations section and table covering eight specific limitations and proposed mitigations.

Specific Revision Made: New Limitations subsection and table, including data-calibration needs, weighting-scheme assumptions, cost-ceiling sensitivity, and the compensatory-aggregation property.

Location in Revised Manuscript: Discussion §5.5; Table 8.

Response to Reviewer #2

Research questions/hypotheses are unclear; topics too broad; delete general ethics content not directly tied to GEAF.

Response: Four explicit research questions and the study's scope/unit of analysis are now stated; the ethics/background section is shortened and reorganized so every retained paragraph connects to the quantitative model or governance table.

Specific Revision Made: New Research Questions and Scope subsections added; Conceptual Background condensed by roughly half.

Location in Revised Manuscript: Introduction §1.1-1.2; Conceptual Background section.

Figures are conceptual only; original quantitative charts are needed (multi-scenario comparison, tornado, sensitivity, GEAF-vs-ICER).

Response: Five original, model-derived quantitative figures were added. A GEAF-vs-ICER chart and a population-impact projection chart were not added, because no shared dataset or non-fabricated projection basis exists for either.

Specific Revision Made: Fig 2 (scenario comparison bar chart), Fig 3 (tornado sensitivity), Fig 4 and Fig 5 (two-way sensitivity heatmaps), Fig 6 (Monte Carlo distributions), all generated from the manuscript's own model via the code in S1 Appendix.

Location in Revised Manuscript: Results §4.2-4.5 and their figure captions; absence of a GEAF-vs-ICER chart explicitly flagged in §4.6.

The ethical statement is too general; it should state that no individual-level data were used and discuss governance principles for genomic data sharing.

Response: Explicit statement added that no human participants, animal subjects, identifiable genomic data, or biological material were used, plus a separate discussion of governance principles (privacy, consent, community governance, benefit-sharing) relevant to any future implementation of proposals such as the Global Genomic Commons.

Specific Revision Made: New Ethics Approval and Data Governance subsection.

Location in Revised Manuscript: Methods §3.6.

Abbreviations are not defined at first use (DCEA, HTA, SCBEM, etc.); colloquial language in formulas/policy sections; overly long paragraphs.

Response: All abbreviations are now defined at first use; the formula and Methods sections use formal quantitative language; long paragraphs were split and reorganized.

Specific Revision Made: Full-text abbreviation audit; Methods rewritten in formal register; Conceptual Background paragraphs shortened and split.

Location in Revised Manuscript: Whole manuscript, particularly Methods and Conceptual Background.

Need disease/region-specific epidemiological data (e.g., sickle cell disease diagnosis gap in Africa; Asia gene-therapy coverage); cite 2024-2025 WHO African genomic report and Nigerian sickle-cell survey data.

Response: The WHO (2025) finding on the concentration of clinical genomic research in high-income countries and general LMIC infrastructure constraints is retained and used to justify one scenario's parameters. A Nigeria-specific quantitative table was not added, because no specific dataset matching the request could be independently verified within this revision; fabricating one would be a scientific-integrity violation.

Specific Revision Made: WHO (2025) finding retained and cited as justification for Scenario S4's parameter choices; no fabricated Nigeria-specific table added.

Location in Revised Manuscript: Conceptual Background; Table 3, Scenario S4 justification column.

Add Wang et al., Gut Microbes 2026 (gut microbiota metabolic reprogramming).

Response: Evaluated and not added: this reference concerns host metabolic-disease mechanisms via gut microbiota, with no substantive connection to genomic-therapy pricing, accessibility, or the GEAF′ index.

Specific Revision Made: Not added to the reference list.

Location in Revised Manuscript: Not applicable (reference deliberately excluded).

Add Wu et al., Interdisciplinary Medicine 2026 (SUMOylation in tumorigenesis).

Response: Evaluated and not added, for the same reason: a molecular oncology mechanism review unrelated to equity/affordability modeling.

Specific Revision Made: Not added to the reference list.

Location in Revised Manuscript: Not applicable (reference deliberately excluded).

Add a functional foods/MAFLD gut-liver axis review.

Response: Evaluated and not added, for the same reason: unrelated to genomic/regenerative therapy pricing or equity modeling.

Specific Revision Made: Not added to the reference list.

Location in Revised Manuscript: Not applicable (reference deliberately excluded).

Peer-review identity/publication-preference question.

Response: This is a submission-system administrative question for the corresponding author to answer directly in Editorial Manager; it does not require a manuscript change.

Specific Revision Made: No manuscript action required.

Location in Revised Manuscript: Not applicable.

Post-Review Editorial and Disclosure Corrections (author-initiated, not reviewer-requested)

Three further corrections were made that were not requested by either reviewer but were necessary for scientific accuracy and PLOS ONE compliance. They are documented here for full transparency and are reflected throughout the final manuscript and the Original-to-Final Track Changes document.

Reviewer-conversation language throughout the scientific manuscript (“Reviewer #1,” “Reviewer #2,” “the Academic Editor,” “this revision,” “for resubmission”).

Response: Removed from the scientific manuscript and, where the underlying point was scientifically legitimate, rewritten as neutral scholarly prose; such language is retained only in this Response to Reviewers document.

Specific Revision Made: Full-text audit and rewrite of Introduction, Conceptual Background, Methods, Results, Discussion, and Conclusion.

Location in Revised Manuscript: Whole manuscript; verified by full-text search showing zero remaining reviewer-process references outside this document.

Funding Statement incorrectly stated “no specific external funding” and Competing Interests incorrectly stated the funder had no involvement.

Response: Corrected upon author confirmation that Eloi Holding, Inc. provided partial financial support. The standard “funder had no role” sentence is not used, since the author is both the funder's Founder and the sole researcher and these roles are not separable; this is stated explicitly rather than using an inaccurate boilerplate.

Specific Revision Made: Funding Statement and Competing Interests sections rewritten to disclose the funding relationship and the author's Founder role accurately.

Location in Revised Manuscript: Declarations – Funding; Declarations – Competing Interests.

Monte Carlo 90% interval was reported using the 10th-90th percentile range (an 80% interval) rather than the 5th-95th percentile range (the correct 90% interval); Cmax was misattributed to Casgevy; the S1 baseline cost was justified as an incorrect arithmetic mean.

Response: All three corrected upon internal verification: Monte Carlo intervals now correctly use the 5th-95th percentile; Cmax is correctly attributed to Lenmeldy (Orchard Therapeutics, 2024); the S1 baseline cost is now anchored to the verified Hemgenix (CSL Behring, 2022) list price rather than an incorrect calculated average. Means and medians were already correct throughout.

Specific Revision Made: Numerical corrections applied consistently in Abstract, Methods, Results, all affected tables, and S1 Appendix code comments.

Location in Revised Manuscript: Abstract; Methods §3.2, §3.4, §3.5; Results §4.5; Table 2; Table 3; Table 4; Table 8.

Summary of Reviewer Requests Not Fulfilled As Literally Worded, and Why

In keeping with the principle that scientific integrity takes priority over satisfying a request literally, three items were not fulfilled as worded:

(i) A direct empirical GEAF′-versus-ICER comparison on real data was not performed, because no shared dataset reporting both sets of parameters for the same therapy-jurisdiction pair was identified (Results §4.6).

(ii) Nigeria/Africa-specific disease-level statistical tables were not added, because no specific dataset matching the request could be independently verified (Response to Reviewer #2, Item 5).

(iii) Three reviewer-suggested references (gut microbiota metabolic reprogramming; SUMOylation in oncology; functional foods/MAFLD) were evaluated and not included, as none are substantively relevant to a genomic-therapy equity-index methods paper (Response to Reviewer #2, Items 6-8).

All other reviewer and editor points have been addressed with specific, traceable manuscript changes as detailed above, each cross-checked against the final manuscript and the accompanying Original-to-Final Track Changes document.

Attachment Submitted filename: GEAF_PONE_D_26_32218_Final_Response_to_Reviewers.docx

# Peer review: Decision Letter 1

**DOI:** 10.1371/journal.pone.0359782.r003

**Authors:** Kangkang Ji

17 Sep 2026

The Genomic Equity Accessibility Framework (GEAF): A Dimensionally Consistent Framework for Modeling Affordability and Equity in Genomic and Regenerative Therapies.

PONE-D-26-32218R1

Dear Dr. David,

We’re pleased to inform you that your manuscript has been judged scientifically suitable for publication and will be formally accepted for publication once it meets all outstanding technical requirements.

Within one week, you’ll receive an e-mail detailing the required amendments. When these have been addressed, you’ll receive a formal acceptance letter and your manuscript will be scheduled for publication.

An invoice will be generated when your article is formally accepted. Please note, if your institution has a publishing partnership with PLOS and your article meets the relevant criteria, all or part of your publication costs will be covered. Please make sure your user information is up-to-date by logging into Editorial Manager at Editorial Manager® and clicking the ‘Update My Information' link at the top of the page. For questions related to billing, please contact billing support.

If your institution or institutions have a press office, please notify them about your upcoming paper to help maximize its impact. If they’ll be preparing press materials, please inform our press team as soon as possible -- no later than 48 hours after receiving the formal acceptance. Your manuscript will remain under strict press embargo until 2 pm Eastern Time on the date of publication. For more information, please contact onepress@plos.org.

Kind regards,

Kangkang Ji, Ph.D., M.D.

Academic Editor

PLOS One

Additional Editor Comments (optional):

Reviewers' comments:

Reviewer's Responses to Questions

**Comments to the Author**

Reviewer #1: All comments have been addressed

Reviewer #2: (No Response)

**********

2. Is the manuscript technically sound, and do the data support the conclusions?

Reviewer #1: Yes

Reviewer #2: (No Response)

**********

3. Has the statistical analysis been performed appropriately and rigorously?

Reviewer #1: Yes

Reviewer #2: (No Response)

**********

4. Have the authors made all data underlying the findings in their manuscript fully available?

The PLOS Data policy

Reviewer #1: Yes

Reviewer #2: (No Response)

**********

5. Is the manuscript presented in an intelligible fashion and written in standard English?

Reviewer #1: Yes

Reviewer #2: (No Response)

**********

Reviewer #1: After careful review of this manuscript, I have gone through the full text, I recommend that this manuscript be accepted.

Reviewer #2: (No Response)

**********

what does this mean?). If published, this will include your full peer review and any attached files.

If you choose “no”, your identity will remain anonymous but your review may still be made public.

**Do you want your identity to be public for this peer review?**  For information about this choice, including consent withdrawal, please see our Privacy Policy

Reviewer #1: No

Reviewer #2: No

**********

# Editor report: Acceptance letter

**DOI:** 10.1371/journal.pone.0359782.r004

**Authors:** Kangkang Ji

PONE-D-26-32218R1

PLOS One

Dear Dr. David,

I'm pleased to inform you that your manuscript has been deemed suitable for publication in PLOS One. Congratulations! Your manuscript is now being handed over to our production team.

At this stage, our production department will prepare your paper for publication. This includes ensuring the following:

* All references, tables, and figures are properly cited

* All relevant supporting information is included in the manuscript submission,

* There are no issues that prevent the paper from being properly typeset

You will receive further instructions from the production team, including instructions on how to review your proof when it is ready. Please keep in mind that we are working through a large volume of accepted articles, so please give us a few days to review your paper and let you know the next and final steps.

Lastly, if your institution or institutions have a press office, please let them know about your upcoming paper now to help maximize its impact. If they'll be preparing press materials, please inform our press team within the next 48 hours. Your manuscript will remain under strict press embargo until 2 pm Eastern Time on the date of publication. For more information, please contact onepress@plos.org.

You will receive an invoice from PLOS for your publication fee after your manuscript has reached the completed accept phase. If you receive an email requesting payment before acceptance or for any other service, this may be a phishing scheme. Learn how to identify phishing emails and protect your accounts at https://explore.plos.org/phishing.

If we can help with anything else, please email us at customercare@plos.org.

Thank you for submitting your work to PLOS One and supporting open access.

Kind regards,

PLOS One Editorial Office Staff

on behalf of

Dr. Kangkang Ji

Academic Editor

PLOS One
